# StabLyzeGraph: High‐throughput screening of combinatorial mutations using graph neural networks

**DOI:** 10.1002/pro.70534

**Published:** 2026-03-07

**Authors:** Muhammad Waqas, Benito Natale, Michele Roggia, Prasenjit Prasad Saha, Mauro Mileni, Sandro Cosconati

**Affiliations:** ^1^ DiSTABiF University of Campania Luigi Vanvitelli Caserta Italy; ^2^ Abilita Therapeutics Inc. San Diego California USA

**Keywords:** computational tool, graph neural networks, mutation analysis, protein engineering, protein stability

## Abstract

Engineering protein stability is a powerful strategy across biotechnology and medicine, supporting a broad range of applications such as atomic structure determination, discovery of therapeutic molecules, biomanufacturing, diagnostic reagents, industrial biocatalysis, etc. However, achieving rapid and significant improvements has been historically challenging due to the vast mutational space and the complex interplay of sequence, structure, and function. Indeed, traditional experimental and computational methods often struggle to predict the impact of multiple mutations and effectively integrate diverse data types. To address these limitations, we developed StabLyzeGraph, a novel computational framework powered by Graph Neural Networks (GNNs) for protein mutational analysis and classification of stabilizing mutations. StabLyzeGraph represents proteins as graphs, integrating amino acid physicochemical properties, evolutionary conservation scores, and mapped three‐dimensional structural information. The framework consists of a Benchmarking module to evaluate performance, and a Screening module to identify and rank impactful mutations. Benchmarking across 23 diverse datasets demonstrated strong predictive performance, highlighting the GNN's ability to leverage integrated features. Mutational analysis enables the generation and probability scoring of single‐ and multi‐site mutants, demonstrating the model's capacity to classify beneficial combinations of mutants based on learned structural impact rather than mere mutation frequency. StabLyzeGraph also features a user‐friendly Graphical User Interface and demonstrates reasonable computational efficiency and scalability for exploring mutational landscapes. This tool provides a robust and versatile approach to accelerate the efficient discovery of stabilizing mutations with tailored properties and represents a step forward in rational protein design, poised to accelerate the creation of novel biologics with enhanced performance. StabLyzeGraph is freely available on GitHub (https://github.com/cosconatilab/StabLyzeGraph) as an open‐source tool.

## 1 INTRODUCTION

Proteins the fundamental pillars of life, drive nearly every biological process, acting as crucial regulators, enzymes, and receptors. Maintaining their structural stability is paramount, as even minor instability can lead to a catastrophic loss of function in cells (Varadi & Velankar, 2023; Wan et al., 2020). In addition, securing protein stability through engineering is a critical need with important implications across biotechnology and medicine (Asial et al., 2013). However, protein engineering is a formidable challenge, demanding thorough exploration of a vast combinatorial space of mutations while carefully considering the intricate interplay between amino acid sequence, folding dynamics, three‐dimensional (3D) structure, intrinsic protein plasticity, and ultimately, function (Calland, 2003; Lutz, 2010). Thus, predicting whether a given mutation will be beneficial or destabilizing remains a significant bottleneck in the field.

Traditional protein engineering approaches heavily rely on experimental methods like site‐directed mutagenesis and high‐throughput screening (Fisher & Pei, 1997; Wójcik et al., 2015). While valuable, these methods are often laborious, time‐consuming, and expensive. On the other hand, computational tools offer a promising alternative, and software like FoldX (Schymkowitz et al., 2005), Rasp (Blaabjerg et al., 2023), Rosetta‐ddG (Sora et al., 2023), PoPMuSIC (Dehouck et al., 2011), IMutant3 (Marabotti et al., 2021), and DFire (Zhou & Zhou, 2002) help predict the impact of point mutations on protein stability. However, these widely used computational tools might be limited by their inability to consider the intricate interplay of multiple mutations, and they lack the scalability needed to explore a vast mutational landscape. Furthermore, their predictive power can be compromised when applied to proteins with limited annotation data, hindering their broader applicability (Navarro & Ventura, 2022).

Machine‐learning (ML) powered tools are nowadays being used for predicting the impact of mutations on protein stability. In this context, deep mutational scanning (DMS) offers a large‐scale experimental dataset that serves as a benchmark for model validation. In fact, in a study on human myoglobin, Kung et al. demonstrated strong concordance between DMS‐derived stability data and ML predictions, while also uncovering stabilizing mutations, including a novel disulfide bond with >17°C thermostability gain (Küng et al., 2025).

The advent of ML, particularly graph neural networks (GNNs), offers a powerful new avenue for addressing these limitations. GNNs excel at representing structured biological data, such as proteins, by capturing complex interdependencies between amino acids (Fasoulis et al., 2021). Their ability to model protein sequences while embedding physicochemical properties allows for the representation of 3D protein structures, even with limited data (Muzio et al., 2021). In GNN‐based protein graph representations, protein residues can be represented as nodes, and edges encode the biochemical and evolutionary relationships between them. By learning from these interconnected features, GNNs demonstrate a superior ability to predict the effects of mutations on proteins with high precision (Gogoshin & Rodin, 2023).

In this work, we have developed a computational framework, called StabLyzeGraph, which utilizes the encoding power and predictive strength of GNNs. This approach seeks to improve existing protein engineering methods by enabling the prediction of mutations that enhance protein stability, even in intricate multi‐mutational scenarios between natural amino acids. The StabLyzeGraph's framework comprises two core modules: Module I, “Benchmarking,” which rigorously evaluates the tool's predictive performance across diverse datasets (from now on, this term specifically refers to a specific collection of active and inactive mutants derived from a single protein), and Module II, “Screening or Mutation Analysis,” which identifies and prioritizes impactful mutations using the trained models. Our approach employs graph‐based representations to capture the subtle interplay between protein sequence, structure, and stability. Additionally, a user‐friendly Graphical User Interface (GUI) is provided, which eliminates the need for Command Line Interface (CLI) expertise. This enables researchers from diverse backgrounds who are not comfortable CLIs to exploit this technology and contribute their part in protein engineering by identifying candidate mutations with the potential to improve protein stability.

Recent tools such as ThermoMPNN (Dieckhaus et al., 2024), Stability Oracle (Diaz et al., 2024), mutDDG‐SSM (Li et al., 2024), ProstaNet (Liang et al., 2025), and UniMutStab (Li et al., 2025) have substantially advanced the prediction of protein stability changes upon mutation by leveraging deep learning architectures and protein language models. Nonetheless, these approaches predominantly emphasize single‐point ΔΔ*G* regression tasks and frequently rely on pre‐trained or transfer‐learning‐based embeddings. In contrast, StabLyzeGraph is specifically engineered to model multi‐site combinatorial effects through direct, graph‐based integration of physicochemical, evolutionary, and structural features. Furthermore, its classification‐oriented formulation and intuitive graphical interface provide an interpretable and accessible framework for large‐scale, high‐throughput stability screening, thereby extending beyond the functional scope of existing methodologies.

Benchmarking results across 20 selected datasets demonstrated strong performance with an overall mean of receiver operating characteristic area under the curve (ROC AUC) of 0.932, precision‐recall area under the curve (PRC AUC) of 0.910, F1‐score of 0.901, Matthew Correlation Coefficient (MCC) of 0.751. All in all, this study introduces a powerful and versatile tool conceived to streamline protein engineering, accelerating the discovery of stable proteins with tailored properties for a wide range of applications.

## 2 RESULTS

### 2.1 Benchmarking GNN model reveals sensitivity to protein length, and class balance

The benchmarking analysis of the 20 datasets revealed that their diverse sequence lengths and distributions played a key role in determining the model's performance and the utility of GNN‐based features (Table 1). Analysis of Dataset 6 shows that the model achieves a high precision score of 0.987, showcasing its strong ability to correctly classify active sequences, with very few false positives returned (0.005). The recall score of 0.975 indicates that the model captures a good portion of active sequences, while the F1‐score of 0.987 strikes a solid balance between precision and recall, indicating the model's general ability to perform well, without favoring one class over the other. The ROC AUC is 0.998, and the PRC AUC is 0.997, both highlighting and further confirming the model's ability to distinguish between the active and inactive classes. The MCC of 0.974 confirms the model's excellent quality, indicating a near‐perfect correlation between predictions and actual labels. By equally weighing all four components of the confusion matrix, MCC works particularly well when dealing with imbalanced class distributions (a scenario quite recurrent in real‐life screening) by offering a reliable evaluation where other metrics might be misleading.

**TABLE 1:** Benchmarking results of datasets: Statistical evaluations for all datasets are presented for model performance metrics (i.e., precision, recall, false positive rate [FPR], F1 score, Mathew Correlation Coefficient [MCC] and area under the curve [AUC]). Metrics are the mean over a five‐fold cross‐validation scheme.

Datasets	Optimal threshold	Precision	Recall	FPR	F1 score	ROC AUC	PRC AUC	MCC
Dataset 1	0.99	1.00	0.93	0.00	1.00	1.00	1.00	0.96
Dataset 2	0.74	0.74	0.87	0.13	0.74	0.93	0.78	0.72
Dataset 3	0.88	1.00	0.60	0.00	1.00	1.00	1.00	0.74
Dataset 4	0.63	1.00	0.91	0.00	1.00	1.00	1.00	0.89
Dataset 5	0.73	0.98	0.92	0.01	0.98	1.00	1.00	0.92
Dataset 6	0.89	0.99	0.97	0.01	0.99	1.00	1.00	0.97
Dataset 7	0.90	1.00	0.84	0.00	1.00	1.00	1.00	0.89
Dataset 8	0.74	1.00	0.93	0.00	1.00	1.00	1.00	0.82
Dataset 12	0.78	0.49	0.76	0.32	0.49	0.70	0.49	0.43
Dataset 16	0.67	1.00	0.57	0.00	1.00	1.00	1.00	0.73
Dataset 17	0.76	0.95	0.70	0.07	0.95	0.90	0.92	0.65
Dataset 18	0.82	1.00	0.75	0.00	1.00	1.00	1.00	0.84
Dataset 19	0.64	0.72	0.63	0.23	0.72	0.70	0.69	0.44
Dataset 21	0.95	0.97	0.94	0.02	0.97	0.98	0.96	0.92
Dataset 23	0.65	0.93	0.81	0.06	0.93	0.93	0.95	0.76
Dataset P00644	0.48	0.67	0.63	0.27	0.67	0.75	0.75	0.38
Dataset P00648	0.64	0.69	0.70	0.18	0.69	0.83	0.76	0.53
Dataset P00720	0.57	1.00	0.93	0.00	1.00	1.00	1.00	0.96
Dataset 5H2C	0.43	0.74	0.87	0.13	0.74	0.93	0.78	0.72
Dataset ADGRL1	0.49	1.00	0.60	0.00	1.00	1.00	1.00	0.74

Abbreviations: ROC AUC, receiver operating characteristic area under the curve, PRC AUC; precision‐recall area under the curve.

**FIGURE 1:**
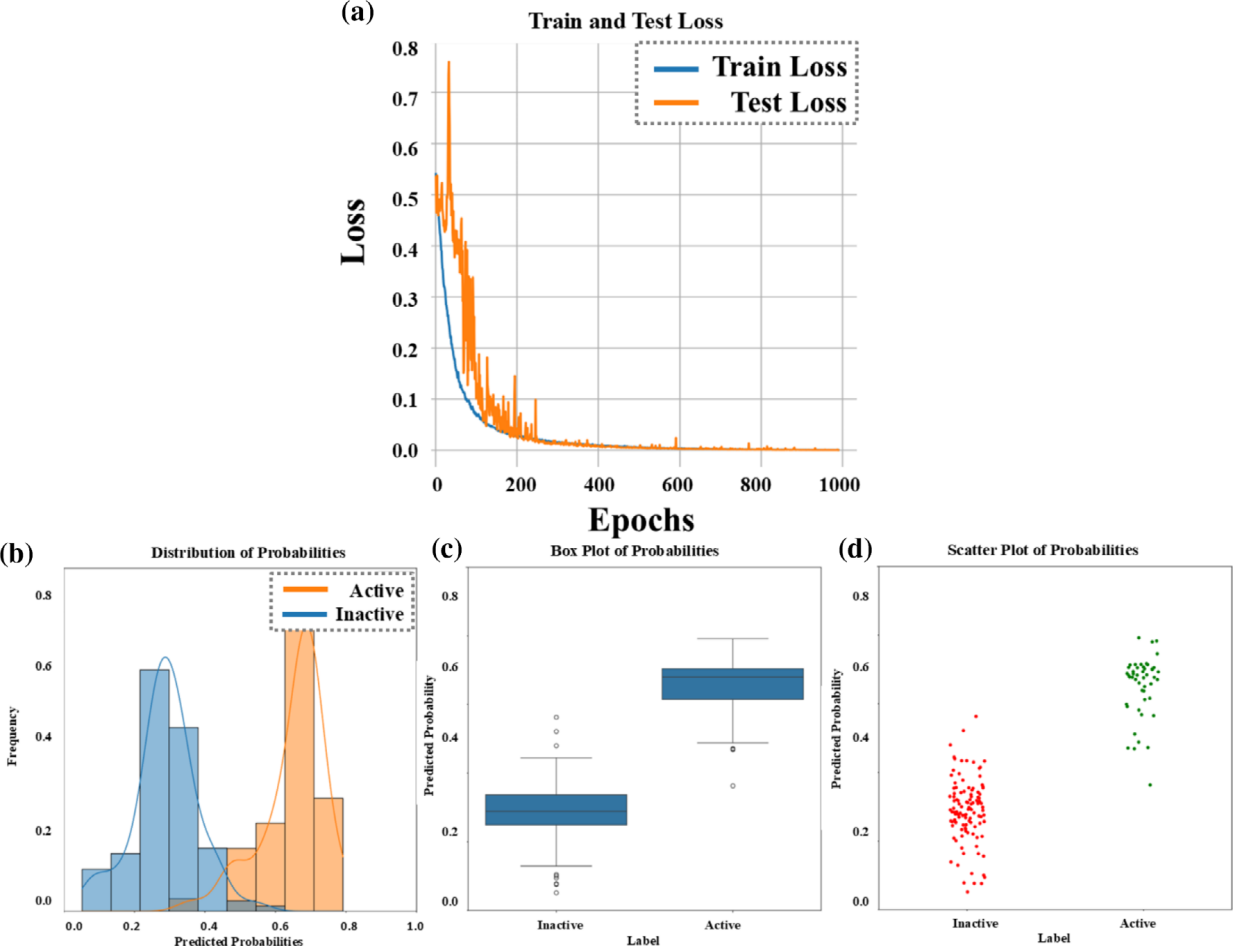
Benchmarking Dataset 6: (a) *training and testing loss*: the training loss is shown in blue, and the testing loss in orange. (b) *Probability distribution*: A histogram visualizes the distribution of inactive (blue) and active (orange) class probabilities. (c) *Probability box plot*: This plot demonstrates the clear separation and balanced prediction between active and inactive classes. (d) *Probability scatter plot*: This scatter plot illustrates the confidence levels for both active (green) and inactive (orange) probability predictions.

For the training phase, 128 hidden units for all network layers, a dropout rate of 0.2, and a learning rate of 0.00001 were used, alongside an L2 regularization set at 0.001. Model evaluations and hyperparameters for all datasets can be seen in Supporting Information Data 1. These choices helped reduce overfitting, and the model showed steady improvement, with a consistent decrease in training and validation loss over the epochs (Figure 1a). By evaluating the fluctuations in loss curves, it is observed that the test loss curve exhibits higher volatility when compared to the train loss one. However, despite these fluctuations in the first part of the training phase, the model is indeed learning and able to generalize as indicated by the consistent decrease in the test loss matching the training loss decreasing trend. Also, early stopping was used with a patience of 50 epochs, to prevent unnecessary training and potential overfitting problems once the model reached convergence.

Figure 1b histogram illustrates the distributions of predicted probabilities for the active and inactive classes. The active class (orange) is predominantly concentrated at high probabilities, indicating the model's strong confidence in correctly classifying the positive class. The inactive class (blue) also demonstrates a high level of model certainty. A clear separation between the active and inactive classes is evident from examining both distributions (Figure 1b). This is somehow desired to better evaluate StabLyzeGraph's generalizability power, as the model will be dealing with highly similar data. The distinction between active and inactive classes is also further substantiated by a box plot diagram (Figure 1c), which shows inactive sequences with probabilities tightly clustered around 0.25, indicating consistent prediction. In contrast, active sequences exhibit a wider probability distribution, predominantly centered around 0.6. Visual reinforcement of these findings is provided by a scatter plot (Figure 1d), where active sequences on the top (green dots) primarily occupy the higher probability region. Conversely, inactive sequences (red dots) are generally found in the region corresponding to probabilities around 0.20–0.35 (typically), affirming the model's effective differentiation between the classes. In summary, results from Dataset 6 demonstrate the model's good capability in classifying protein sequences based on structural and physicochemical features. The combination of high precision, recall, and F1‐score, along with strong AUC values (see Table 1), proves the model's reliability in distinguishing between the active and inactive classes. The visualizations also highlight the clear separation between the two classes, reinforcing the model's accuracy (Narayanan et al., 2021).

The GNN features seem to have a consistent positive impact on model performance across all datasets. A performance comparison without GNN‐constructed features is provided in Supporting Information Data 6, confirming the key role of the GNN‐constructed features on the model performance. However, the effectiveness of these integrated graph‐based features depends on different aspects of the considered datasets, that is, protein sequence length, mutation distribution, and size of the datasets. These factors play a crucial role in the determination of the model's capability to learn and generalize: in fact, shorter sequences might lack sufficient structural complexity for the model to learn the spatial interconnections between residues, while an imbalanced mutational distribution (data skewed toward only a few residue positions) or a low mutational landscape (limited number of unique mutation sites) could limit the model's performance. So, GNNs leverage these sparse datatypes and merge them to get a richer and more informative training source. These observations describe the strict dependency between dataset properties and the effective performance of the predictive models.

The comprehensive evaluation of all datasets indicates that the predictive performance of the StabLyzeGraph model is governed not by dataset size or protein length but by the intrinsic quality of the data. Specifically, the model's reliability depends on the conservation, nature, and structural coherence of mutations rather than on purely quantitative attributes. Datasets characterized by high conservation and structurally consistent mutation patterns enabled the model to detect functionally meaningful features with high fidelity (e.g., Dataset 7 has a protein sequence length of 148 amino acids and 21 total unique mutation positions, showing an MCC of 0.89). In contrast, datasets marked by sequence heterogeneity, longer protein chains, or noisy mutational signals exhibited weakened discriminative capacity (e.g., Dataset ADGRL1 with a long protein sequence length of 1515 amino acids, shows an MCC of 0.52).

**FIGURE 2:**
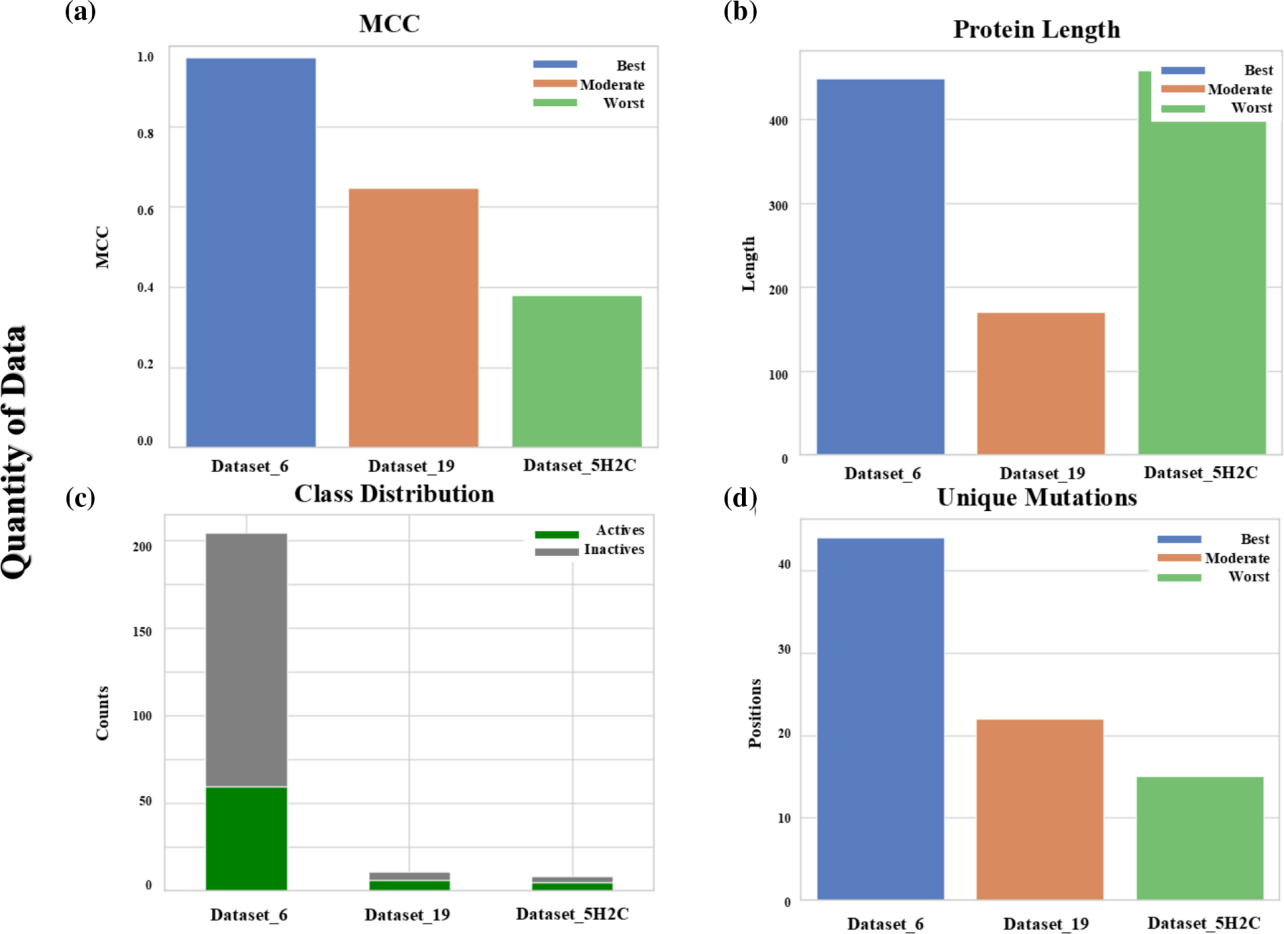
Impact of datasets' composition and nature on model performances: (a) Datasets are classified into three performance groups—best (blue), moderate (orange), and worst (green), based on the Matthews Correlation Coefficient (MCC). (b) Protein lengths are plotted for three representative datasets from each performance group. (c) Class distributions for active and inactive classes are shown for the same three datasets. (d) Unique mutational positions are identified and visualized.

Among the evaluated datasets, Dataset 6 emerged as the top performer, with an MCC of 0.973, a precision of 0.988, and a recall of 0.974, comprising 239 active and 579 inactive variants. This remarkable outcome reflects a strong similarity among active variants, where conservative mutations conveyed structurally informative signals that enhanced both model learning and generalization. These findings demonstrate that data quality (and specifically, well‐conserved mutational patterns) can outweigh data quantity in determining predictive success (Figure 2 and Supporting Information Data 2). Similarly, Dataset P00648 (MCC = 0.920) and Dataset 1 (MCC = 0.955) achieved near‐ideal metrics, including F1 and AUC scores of ~1.000. Both datasets share a notable internal structural uniformity, suggesting that conserved mutations within active sites supported the formation of robust feature–label associations, sustaining consistent predictive stability.

Conversely, Dataset 2 (MCC = 0.720), which presents extended protein length (354 residues), introduced structural complexity that diminished the model's interpretability and generalization capacity. In fact, higher false positive rate and modest precision and recall in this dataset suggest that elongated, complex proteins may introduce superfluous sequence features that obscure critical functional patterns. Other datasets in the mid‐performance range, such as Dataset 19 (MCC = 0.647), although relatively well‐balanced in terms of active/inactive mutations ratio, show moderate recall that can indicate that class balance alone cannot guarantee improved performance when mutational variation lacks structural or biochemical significance. In particular, and when compared to the best‐performing Dataset 6, Datasets 2 and 19 smaller areas under the curve values (ROC AUC = 0.927 for Dataset 2, ROC AUC = 0.895 for Dataset 19) further illustrate the concept that stable prediction requires both consistent sequence context as well as meaningful biochemical descriptors.

At the opposite end of the spectrum, Dataset 5H2C demonstrated very weak performance (MCC = 0.379), being characterized by a modest recall (0.633) and a high false positive rate (0.267) across a small sample of 19 actives and 14 inactives (458 residues). The dataset's poor conservation of functional residues, as well as its limited diversity, likely impaired the model's capacity to identify meaningful classification boundaries. Dataset ADGRL1 (MCC = 0.526), with extended protein length of 1515 residues, also failed to perform better. Similarly, Dataset 16, showing low MCC and AUC values, suffered from noisy or inconsistent mutational profiles that hindered the learning of reliable physicochemical patterns (Supporting Information Data 2).

Overall, this comparative analysis highlights that larger datasets and/or longer protein sequences do not inherently translate into superior model performance. Instead, the StabLyzeGraph model demonstrates predictive accuracy and stability when trained on datasets with high‐quality, mutation‐conserved, and structurally coherent features. These findings emphasize that data homogeneity and functional relevance, rather than quantitative scale, are the principal determinants of the model's predictive power and generalization capability.

To validate the predictive capability of StabLyzeGraph against established physics‐based methods, we performed a direct benchmarking comparison with ThermoMPNN (Dieckhaus et al., 2024). To ensure a rigorous and diverse evaluation, we selected four datasets representing distinct protein families, varying sequence lengths, and differing data densities: the community‐standard T4 Lysozyme (Dataset 1), our primary case study Lysozyme C (Dataset 7), the densely sampled Protein G (Dataset 6), and the structurally distinct Thermonuclease (Dataset P00644). To ensure a fair comparison independent of arbitrary decision thresholds, performance was evaluated using the ROC AUC.

ThermoMPNN achieved a robust mean ROC AUC of 0.84 across the four datasets. However, StabLyzeGraph achieved a higher mean ROC AUC of 0.94 on the same targets (see Table 2). Specifically, StabLyzeGraph demonstrated near‐perfect ranking capabilities (ROC AUC ≈ 1.0) on Datasets 1, 6, and 7, significantly outperforming the physics‐based baseline. Conversely, ThermoMPNN performed better on Dataset P00644 (0.84 vs. 0.75), suggesting that for certain targets, the generalist physical potentials may offer complementary advantages to the protein‐specific GNN approach.

**TABLE 2:** Comparison of receiver operating characteristic area under the curve values for StabLyzeGraph and ThermoMPNN across four selected protein mutation datasets.

Dataset	ThermoMPNN	StabLyzeGraph
Dataset 1 (T4 Lysozyme)	0.90	1
Dataset 6 (Protein G)	0.77	0.99
Dataset 7 (Lysozyme C)	0.85	1
Dataset P00644 (Thermonuclease)	0.84	0.75
Mean	0.84	0.94

### 2.2 Mutations analysis reveals unbiased stability predictions driven by structural impact

In StabLyzeGraph, theoretical mutants can be generated by three different methods: combinatorial, weighted, and evolutionary (discussed in Section 4), but here we will discuss only mutants generated by the combinatorial method. The decision to focus on the combinatorial method is justified by the need for a broad and unbiased examination of mutation combinations in the dataset, without any external influence from weighting or evolutionary processes. Nevertheless, multiple mutant generation methods were introduced to cater to the user's needs and choice. Other generated mutants can be seen in Supporting Information Data 3. In this work, we considered two mutations per protein sequence for mutant generation, which were then evaluated and ranked based on the predictions made by the selected best‐trained model. A GNN's predicted probability is assigned to each mutant protein for the screening and ranking process. GNN‐based residue‐specific and structural features provided a more biologically relevant predicted stability score based on potential conformational changes induced by these mutations. Specifically, for this analysis, we choose the best performing Dataset 6, comprised of 239 active and 579 inactive mutants (Huang et al., 2020). A total of 4985 generated mutants were generated and then screened using the pretrained GNN model, which predicted F52G:T51A as the most active mutant with a probability score of 0.98611. After analyzing the mutation screening test, the model's impartiality is evaluated, ensuring that it does not favor the most common or frequently occurring mutations but instead predicts the best mutation combinations based on their real impact on protein stability.

**FIGURE 3:**
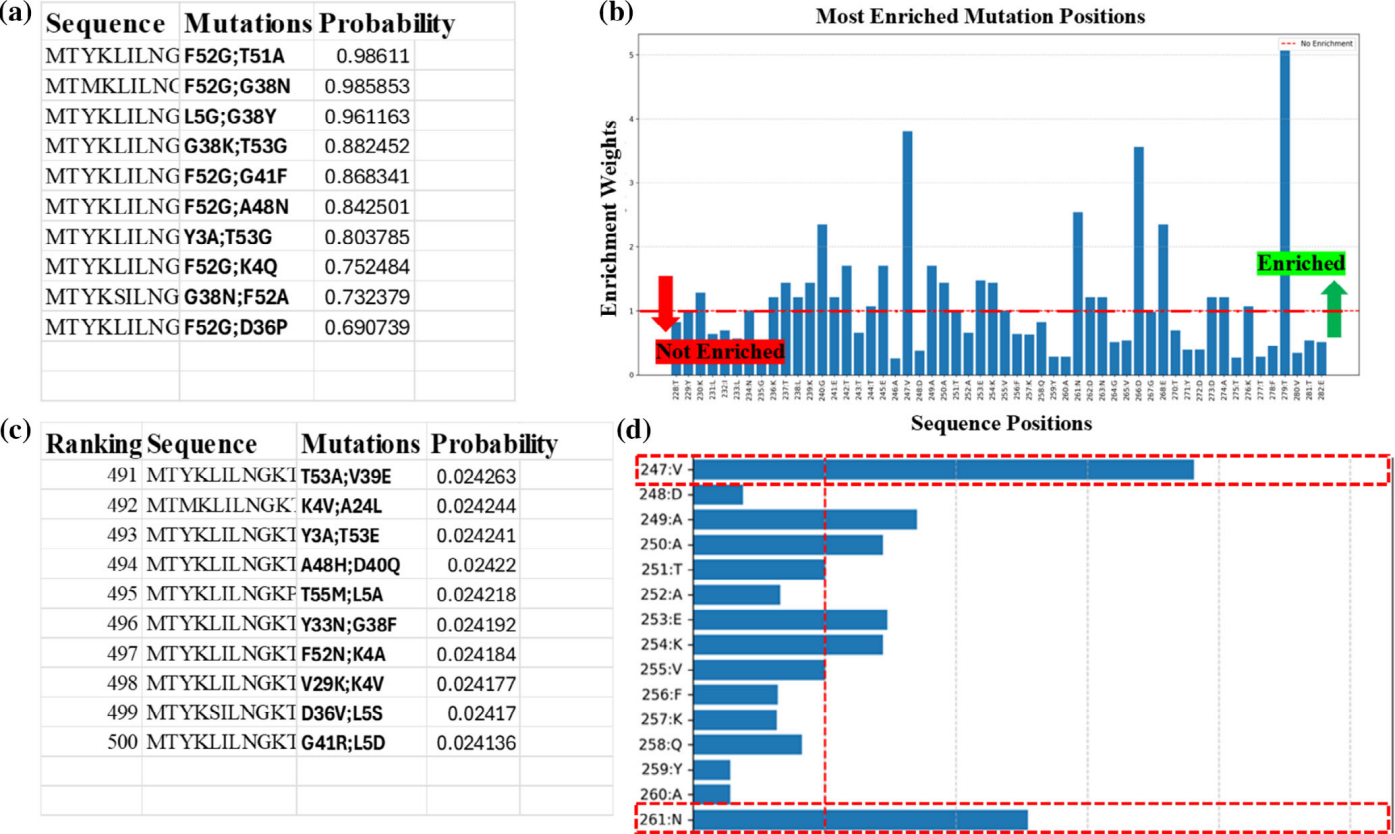
Mutation analysis: (a) Top 10 predicted mutants from mutation analysis module, listing mutant sequence, mutation combinations, and associated predicted probability. (b) Enriched mutation positions are plotted as a bar graph. The most enriched position is T279. (c) Mutants with low probabilities can be seen having multiple mutation combinations having probabilities below 0.02, and (d) two most enriched mutation positions are highlighted in dashed red boxes.

To assess this potential model bias, we specifically examined StabLyzeGraph's predictions for the most enriched mutation positions within Dataset 6. We hypothesized that a model influenced by bias would disproportionately favor these frequently observed positions, predicting them as highly stable. However, our result showed no evidence of such a preference. Notably, two of the most enriched mutational positions (T279 and V247) were not present even in the top 500 predictions (Figure 3). This demonstrates that the model's predictions are not simply driven by the frequency of mutations but are accurately based on the real structural impact learned from the training data patterns. A meaningful distribution of stabilizing and destabilizing mutations suggests that StabLyzeGraph is capturing critical potential protein stability changes. Mutation analysis for Dataset 6 was performed to understand the prediction range of StabLyzeGraph (Supporting Information Data 3 and 4).

### 2.3 Computational efficiency and scalability

Benchmarking and mutation analysis for all datasets were performed to assess computational processing time and memory usage influenced by dataset characteristics (sequence length and number of sequences). It is important to note that the mutation analysis module was significantly slower than the benchmarking process. This is because each mutant must be converted into a protein graph representation to be scored and ranked by the trained model, which is a computationally intensive task. Considering Dataset 7, the mutation analysis took ~0.17 h on a single core and ~0.15 h on 40 cores, but it took ~0.06 and ~0.05 h on a single core and 40 cores, respectively, for the benchmarking experiments while using an Intel® xeon® CPU E5‐2640 2.40 GHz. The time required for mutation analysis is directly proportional to the number of mutants generated, which depends on the number of mutations per mutant specified by the user. In this context, Dataset 7 on a single core took ~1.1 h to generate and rank 4753 mutants, while Dataset 16 on a single core took ~2.1 h for 69,006 mutants (with 40 cores it took ~0.15 and ~0.8 h, respectively), which suggests StabLyzeGraph has scalability, but the processing speed is not linear when considering the total number of mutants being analyzed (the workload) and the overall computational time. Specifically, scalability is well elaborated by comparing the time taken by smaller datasets to the more complex and larger datasets. A runtime summary table regarding this analysis can be seen in Supporting Information Data 5.

## 3 DISCUSSION AND CONCLUSIONS

Our proposed GNN model, namely StabLyzeGraph, addresses the significant challenge of predicting stability‐enhancing mutations in proteins (Cravens et al., 2021; Narayanan et al., 2021). A distinguishing feature of StabLyzeGraph is its capacity to evaluate the collective impact on protein stability when multiple, stabilizing single mutations are introduced concurrently. This is crucial, as the cumulative effect of such combinations is often unpredictable but essential for the identification of optimal mutation sets (Zhou et al., 2024). The GNN framework achieves this goal by integrating diverse data modalities: primary sequence information, from which protein graphs are constructed, alongside amino acid physicochemical properties, 3D mutant structural coordinates mapped onto the wild‐type (WT) structure, and evolutionary conservation scores derived from tools like Clustal Omega (Sievers & Higgins, 2021). This comprehensive approach allows StabLyzeGraph to effectively navigate complex multi‐mutational landscapes and is also advantageous when working with proteins with limited experimental annotation or structural data (Liu & Li, 2023; Nair et al., 2022). StabLyzeGraph demonstrates reasonable scalability, even with limited computational resources. While the benchmarking process itself is faster than the complete mutational analysis (which includes mutant generation and ranking), the tool efficiently handles datasets of varying sizes. For instance, Dataset 7, containing 121 sequences, requires 15 min for benchmarking, whereas the full analysis of mutants takes approximately 1.5 h with multi‐core processing (40 cores) or 6 h on a single CPU. This capability to process larger datasets is vital for exploring extensive mutational landscapes, a time‐complexity relationship well documented in protein studies (Vitorino, 2024; Zhou & Huang, 2024). The integration of 3D coordinates of mutant structures and conservation scores enhances the biological relevance of predictions by reflecting evolutionary relationships and structural context (Gao et al., 2023; Wu et al., 2023).

The performance of StabLyzeGraph is inherently linked to the quality and curation of its training data. To address the inherent imbalance in mutational datasets, where the vast majority of random mutations are deleterious (Romero et al., 2015), we employed a random oversampling approach applied strictly within the training folds during model training. This strategy counteracts the class imbalance by increasing the frequency of minority class patterns during the training phase, without introducing synthetic artifacts that could compromise the biological validity of the graph structures (Yang et al., 2024). Additionally, to support users working with extremely scarce or unbalanced data, the StabLyzeGraph framework specifically proposes a data augmentation module (decoy creation) capable of generating synthetic inactive variants to populate the negative class. To promote wider adoption, a user‐friendly GUI makes the framework accessible to researchers from diverse scientific backgrounds (Willman, 2021).

Our studies also suggest a crucial relationship between the nature of the mutations within a dataset and the efficacy of the StabLyzeGraph model's learning process. Conservative substitutions, which involve replacing an amino acid with one that shares similar physicochemical properties (e.g., size, charge, and hydrophobicity), typically induce subtle chemical and structural perturbations to the protein's folded state and its local environment. These changes, such as minor adjustments in sidechain packing or small shifts in local backbone conformation, are more readily captured by StabLyzeGraph's current feature set, which includes physicochemical properties, evolutionary conservation scores, and spatial relationships derived from 3D coordinates. The graph‐based representation effectively models how these localized changes propagate through the network of residue interactions in the final folded structure.

In contrast, non‐conservative mutations introduce more significant alterations in physicochemical properties. Replacing a small, hydrophobic residue with a large, charged one, for instance, can lead to substantial disruptions of the protein's core packing, alter surface charge distribution, or introduce steric clashes. These changes are not merely localized perturbations, as they have the potential to induce more drastic structural rearrangements, affect protein dynamics, or even influence intermediate states during folding or interactions with cellular machinery (like chaperone factors) that are not explicitly or fully encoded in the current static structural and sequence‐based features. Consequently, deciphering the impact of these larger and potentially global effects presents a greater learning challenge for the model.

Thus, the observed performance landscape across diverse datasets highlights StabLyzeGraph's current proficiency in rapidly discerning patterns within mutational contexts that primarily affect the stability of the final folded protein structure through relatively conserved modifications. This reflects the model's current scope and the type of information that StabLyzeGraph is equipped to process. This insight provides valuable direction for future work, suggesting the need to explore the integration of features specifically tailored to capture the broader range of molecular consequences arising from diverse, particularly non‐conservative, mutational types, including potential impacts on dynamics and interactions beyond the representative folded structure.

All in all, the present work underscores the value of computational methods like StabLyzeGraph in analyzing and leveraging experimental data to potentially improve protein stability. The practical applications of StabLyzeGraph are diverse and impactful within protein engineering. For instance, it could be employed to enhance the thermostability of enzymes used in industrial biocatalysis, leading to more efficient and cost‐effective processes. Similarly, in the development of therapeutic proteins, StabLyzeGraph could guide the engineering of antibodies or other biologics with improved stability and longer half‐life (Chiu et al., 2019). Another promising application lies in bioremediation, where engineered proteins could be designed to degrade pollutants more effectively under harsh environmental conditions (Mohanan et al., 2020). Furthermore, researchers investigating the fundamental principles of protein folding and stability could utilize StabLyzeGraph to explore complex mutational landscapes and gain deeper insights into the factors governing protein stability. This framework could also assist in the design of novel, stable protein building blocks for creating artificial biological systems with tailored functionalities (Larson & Maus, 2021). Importantly, StabLyzeGraph could be adapted for the design of more stable receptors for drug discovery, although this presents unique challenges. Membrane proteins, often targets for drug development, are notoriously difficult to work with due to their complex structures and requirements for specific lipid environments (Stephens & Wilkinson, 2024). Stabilizing these proteins through mutation is a significant hurdle, but StabLyzeGraph, with further development, could potentially navigate these challenges and guide the engineering of membrane proteins with improved stability and suitability for drug screening and development.

While recent models such as ThermoMPNN and mutDDG‐SSM utilize language model or transfer‐learning approaches to estimate single‐site ΔΔ*G* values, StabLyzeGraph expands upon these efforts by introducing a combinatorial, classification‐based framework that directly integrates structural and physicochemical interpretability.

While StabLyzeGraph demonstrates strong performance, certain limitations should be considered. First, the model's performance relies partly on evolutionary conservation scores derived from Multiple Sequence Alignments (MSA). Consequently, its applicability to “orphan proteins” or targets with shallow MSAs (few homologs) may be limited compared to language‐model‐based approaches. Second, while our data augmentation strategies mitigate class imbalance, training on datasets with a very small number of active variants (<100) requires careful cross‐validation to prevent overfitting to sequence‐specific motifs. Finally, as a classification framework, StabLyzeGraph prioritizes the identification of “hits” (screening) rather than the precise quantification of thermodynamic stability (∆∆*G*) or explicit interaction energies.

Future improvements to StabLyzeGraph include developing transfer learning or meta‐learning strategies to take advantage of knowledge across different protein families. This will be pursued alongside incorporating physics‐based constraints to better capture intraresidue interaction changes upon mutation, optimizing computational expenses for larger‐scale analyses, and developing strategies to address the limited availability of high‐quality experimental training data. Furthermore, StabLyzeGraph is currently optimized for the 20 canonical amino acids; the incorporation of non‐natural amino acids remains a significant opportunity for future development as training data becomes available.

## 4 METHODS

### 4.1 Overview of the workflow

**FIGURE 4:**
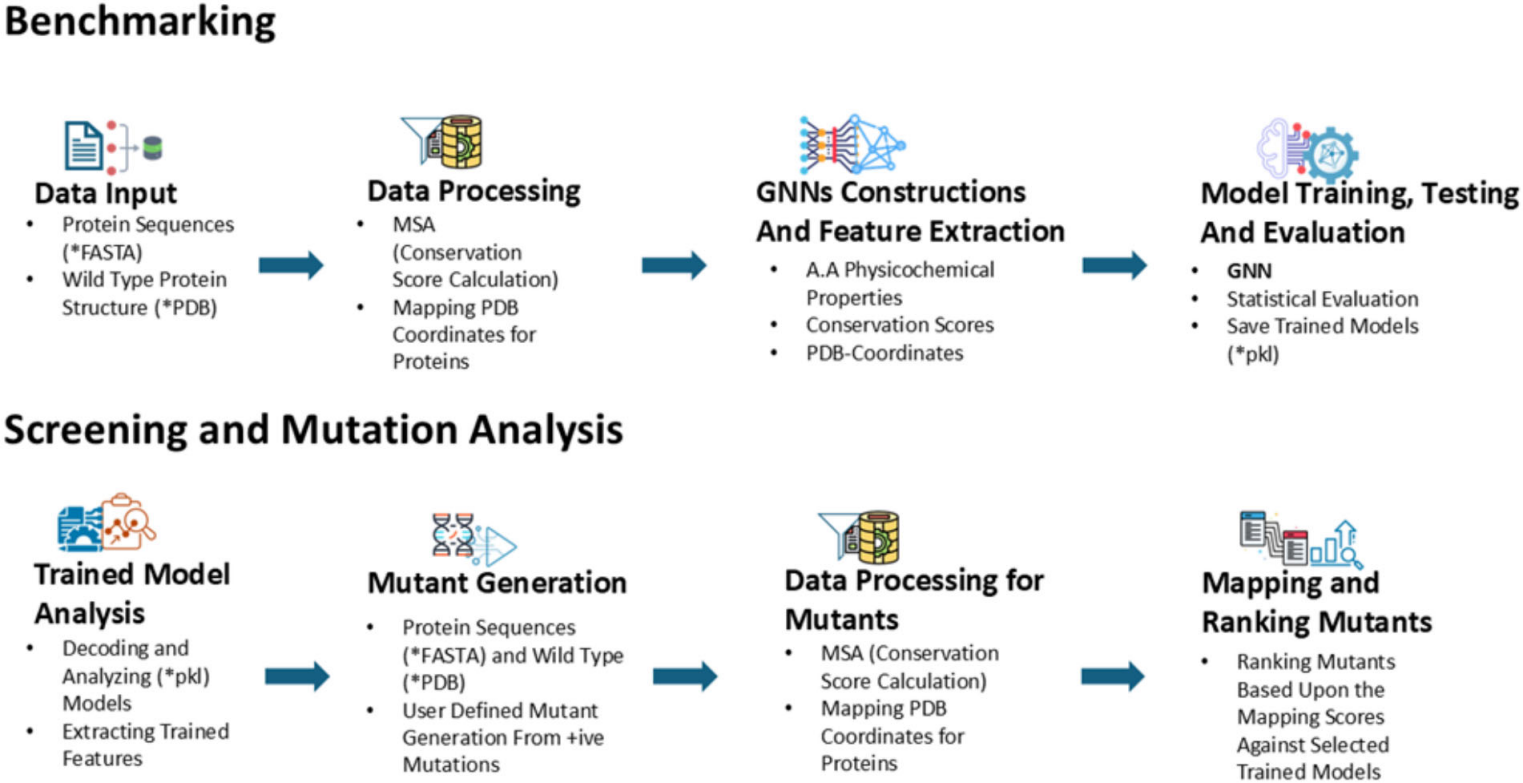
Workflow explanation for StabLyzeGraph: Module I (Benchmarking) starts with data input and processing, which is used for protein graph representation construction. The graph neural networks (GNN) model is trained and evaluated. Subsequently, the selected model is processed during Module II (Mutation Analysis) for mutant generation and ranking. MSA, multiple sequence alignment; PDB, Protein Data Bank.

Our GNN‐powered protein engineering tool integrates a GNN model into a framework where specialized protein graph representations (having three different types of node features) are created for mutational analysis and stability prediction (Gupta et al., 2020). A workflow has been designed (Figure 4), which integrates several computational steps to analyze protein sequences, extract features, predict mutational effects, and identify optimal mutations. It comprises four key stages:

1. *Data input and preprocessing*: Input protein sequences, as well as WT sequence and its 3D structure (here, an experimental or predicted structure can be given as an input Protein Data Bank [PDB] file), are read from the user‐provided files. Sequences are aligned, and conservation scores are computed. Alongside, mutant sequences are mapped using the WT structure as a reference.
2. *Feature extraction and model training using GNNs*: Graph‐based representations of the protein sequences are constructed by incorporating (i) standard physicochemical properties of amino acids, (ii) conservation scores calculated by MSA, and (iii) structural coordinates of protein sequences. After graph construction, GNN is utilized to train and test the model.
3. *Mutant generation and ranking*: Active mutations (i.e., known to be stabilizing or also inducing enrichment of the expression levels) are picked from active protein sequences, and a user‐defined combination is used (how many mutations per mutant) to generate mutants, which are ranked by the trained model.

### 4.2 Data preprocessing

Protein sequences (WT as well as from the more and less stable mutants) are read from FASTA files provided by the user, along with the WT 3D protein structure in PDB format. More stable sequences represent functional variants, less stable ones serve as negative controls, and the WT sequence is used as the baseline for mutation analysis. Standard amino acid properties like hydropathy index, amino acid volume, polarity, and their net charge are provided within StabLyzeGraph, so that protein sequences are automatically encoded accordingly (Ofer et al., 2021). A *Z*‐score normalization is applied to the raw property values, ensuring all features have a mean of 0 and a standard deviation of 1 before being assigned to the nodes. Sequence conservation scores are calculated using MSA by the Clustal Omega Bio‐Python utility (Xue et al., 2009). Specifically, the conservation score for each residue position represents a consensus frequency score: the fraction (ranging from 0.0 to 1.0) of sequences in the alignment that match the consensus amino acid at that given position. Conservation scores set a baseline for the detection of critical residues and regions likely to affect protein stability when mutated. Instead of reconstructing 3D coordinates for each mutant, we map the mutant sequences onto the WT 3D coordinates. This approach is justified because single amino acid mutations generally do not drastically alter protein structures. By utilizing mapped coordinates, we reduce computational complexity while still capturing significant structural information for scoring mutants (Haider, 2021). To deal with the problem of extreme data scarcity, we also propose a data augmentation approach to create a putatively negative mutant from the positive class (a fully detailed explanation is provided in Section 4.5.1). The framework is designed to be robust to missing coordinates within the PDB file by strictly requiring an experimental structure or a predicted model.

### 4.3 GNN construction and model training

Data from the previous preprocessing step are utilized to construct undirected graphs for all protein sequences, which can be mathematically represented by $G=\left(V,E\right)$, where “*G*” is for Graph, “*V*” is the set of all nodes comprising amino acid residues encoded with processed features, and “*E*” denotes the edges of the graphs, which account for the pairwise relationships between residues, incorporating spatial distances from 3D structures or sequence adjacency (José et al., 2014). Graphs are constructed by mapping amino acids to numerical features using built‐in encoding dictionaries. Then, conservation scores calculated during data processing are incorporated along with structural coordinates mapped through the 3D structure of the WT protein, as additional node features. Graph representations account for the spatial atomic connections. Especially, for each amino acid node, an integrated package of information from its 10 Å vicinity is obtained through the construction of edges between nodes within the above‐referenced distance radius. We specifically chose this threshold, as values <10 Å were too strict to get noticeable local interactions, whereas a radius >10 Å was causing too much noise. Edges in the constructed graph are annotated with the Euclidean distance (in Angstroms) between the C‐alpha atoms of the connected nodes. This distance serves as the edge attribute, providing the GNN with spatial context from the 3D coordinates. This geometric information is pivotal for generating node representations that integrate intrinsic node features with details from the surrounding chemical environment (within a 10 Å radius). Thus, by capturing local structural features through this approach, the model develops enriched, context‐aware node representations, furnishing each node with more comprehensive contextual information for effective training. A *K*‐fold cross‐validation strategy (here, with five splits) is applied to yield a more robust estimate of model performance by training and testing on different subsets of the data. This partitioned approach, which relies on disjoint (non‐overlapping) test sets for each fold, provides a structured framework to prevent data leakage.

The Graph Attention Network (GAT) is utilized for graph processing (Zhao et al., 2022). GAT provided more robust attention and supported better weight initialization. The network begins with a Graph Attention Network (Layer) (GATConv) layer that takes input node features of dimension 6 and projects them into a hidden representation, allowing the model to capture local connectivity and feature dependencies among neighboring nodes in the graph. Next, a SAGEConv layer further refines the node embeddings through neighborhood aggregation. This is followed by a Self‐Attention Graph Pooling (SAGPool) layer, which reduces the graph size by learning to discard less important nodes, while preserving essential features. The subsequent global add pooling layer aggregates all features from this reduced graph into a single graph‐level vector representation, providing a comprehensive encoding of the entire protein (Liu et al., 2023). The resulting representation is then passed through fully connected linear layers and a final classification layer for prediction, with dropout applied during training to prevent overfitting.

To address class imbalance, random oversampling was applied exclusively to the training data within each cross‐validation fold. This process involved re‐sampling the indices of the minority class to achieve a balanced distribution for training, while ensuring that the test sets remained original and unmodified to prevent data leakage. The GNN model is then trained on these balanced datasets using conventional training and testing procedures to ensure robustness and optimal parameter selection. Evaluation metrics, including AUC, precision, recall, and F1‐score, are computed to comprehensively assess the model's performance (Naidu et al., 2023).

### 4.4 Mutants generation and rankings

After model evaluation, users can select the best model (as per assessment of the performance metrics computed at the end of the benchmarking phase) to use further for mutation analysis. A detailed mutant library is generated by the user‐defined combination of active sequence mutations (mutations per sequence). Three distinct strategies were implemented for generating mutants, selectable based on user preference: a combinatorial approach, which utilizes all pairs from mutation datasets to create combinations of active mutations; a weighted approach, where an enrichment weight is calculated from the ratio of active versus inactive mutant position frequencies; and an Evolutionary approach, which incorporates an evolutionary algorithm to identify the best‐predicted mutants with higher activity potential by generating new mutants through random selection of parent sequences and single‐point crossover. This iterative process mimicked natural evolutionary processes, enabling the exploration of a broader sequence space. Each mutant sequence is converted to a graph using 3D structural information from a 3D file and amino acid features. Then, these mutants are scored and ranked by the finalized trained model to assess the functional impact of the mutation by getting prediction probabilities. Specifically, this inference uses torch.sigmoid to map raw logits to probabilities of being active. It is worth noting that the calculated score doesn't predict the actual change in stability. Instead, it serves as a predicted probability score comparison to the trained model, inferring the learned patterns from positive and negative classes (Guruprasad et al., 1990; Willman, 2021).

### 4.5 Implementation and benchmarking

#### 4.5.1 Datasets curation

**FIGURE 5:**
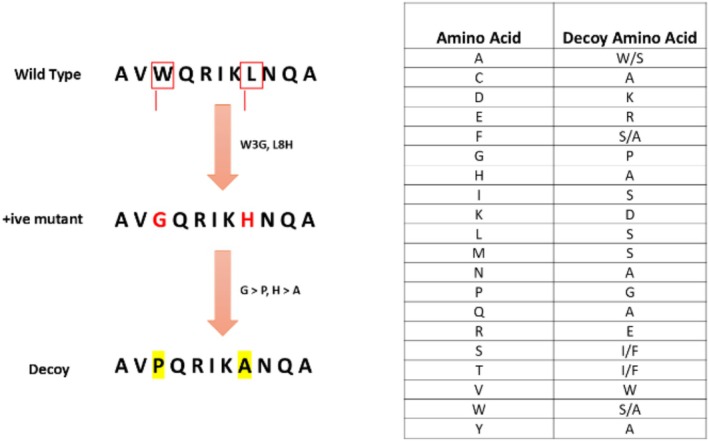
Decoy creation scheme: a wild‐type sequence could be mutated to a positive mutant, which is known to have some improved activity/stability; then a decoy can be created using the opposite amino acid to mimic the active amino acid but opposed to the activity. The table on the right shows the active amino acids and their respective decoy substitutions.

Since we were faced with limited data availability, we selected multiple datasets for StabLyzeGraph implementation and performed benchmarking studies. All datasets were curated to remove redundant sequences and to convert these simple lists into proper FASTA format using our own written Python scripts. To address an extreme imbalanced distribution of active and inactive mutants, we also propose a data augmentation strategy (decoys generation) to create a population of inactive mutants, if needed by the user. While benchmarking was performed using random oversampling to ensure data fidelity, this optional procedure is implemented in the software to assist users with very limited datasets. Specifically, it consists of synthetically generating inactive variants from existing active mutants. The generation process involved introducing specific point mutations into the sequences of active mutants, where amino acid residues were substituted with alternatives exhibiting contrasting physicochemical properties. For example, a negatively charged glutamic acid in an active mutant might be replaced by a positively charged lysine to create a putative inactive mutant with an inverted local charge. Similarly, replacing a hydrophobic residue like phenylalanine with a smaller, polar residue such as serine or a neutral residue like alanine was intended to disrupt local interactions and potentially abolish the phenylalanine stabilizing effect. It is crucial to note that the concept of an “opposite amino acid” is not strictly defined. Therefore, our selection of substitutions was guided by the aim of introducing substantial variations in key properties, namely hydrophobicity, charge, and size, thereby diversifying the dataset of inactive mutants without relying on a rigid definition of amino acid opposition. A scheme on how these decoys could be created is elaborated (Figure 5).

Datasets after curation are presented in Table 3.

**TABLE 3:** Datasets distribution, structure, and sources.

Datasets	Protein family	PDB ID (wild type)	Active/more stable	Inactive/less stable	Sequence length	Source
Dataset 1	Hydrolase	2LZM	75	215	164	See Supporting Informationy Data 7
Dataset 2	Guanine nucleotide‐binding protein G(i) subunit alpha‐1	3UMR	47	302	354	
Dataset 3	Guanyl‐specific ribonuclease T1	1RN1	13	52	130	
Dataset 4	Haloalkane dehalogenase	1MJ5	76	47	302	
Dataset 5	Halohydrin dehalogenase	1PWX	66	89	254	
Dataset 6	Immunoglobulin G‐binding protein G	1PGA	239	579	448	
Dataset 7	Lysozyme C	1LZ1	31	90	148	
Dataset 8	Myoglobin	1BVC	74	14	153	
Dataset 12	Ribonuclease HI	2RN2	42	35	155	
Dataset 16	Thermonuclease	1STN	59	498	231	
Dataset 17	Tryptophan synthase	1WQ5	27	19	268	
Dataset 18	Tyrosine‐protein kinase Fyn	1SHF	12	80	537	
Dataset 19	Flavodoxin	1FTG	24	19	170	
Dataset 21	Dihydrofolate reductase	1RX4	20	75	159	
Dataset 23	Lysozyme C	4LYZ	24	38	147	
Dataset P00644	Thermonuclease	ALPHAFOLD	413	69	231	Dehouck et al. (2011)
Dataset P00648	Ribonuclease	ALPHAFOLD	136	17	157	
Dataset P00720	Muramidase	ALPHAFOLD	67	31	164	
Dataset 5H2C	GPCR	ALPHAFOLD	19	14	458	Wang et al. (2019)
Dataset ADGRL1	GPCR	ALPHAFOLD	51	15	1515	

Abbreviation: PDB, Protein Data Bank.

#### 4.5.2 Benchmarking and performance evaluation

To assess the computation speed, StabLyzeGraph was used on all previously curated datasets by comparing the time taken by the benchmarking module and the mutation analysis module. Also, the time and computational resource complexity were taken into consideration to better understand the relation between performance and dataset distribution. For performance assessment, all datasets were used, and the obtained model performance was evaluated by analyzing the AUCs, *F*‐score, and other statistical measures.

A step‐by‐step “how‐to” guide to run StabLyzeGraph is discussed below.

Before you begin, make sure you have installed StabLyzeGraph as per the instructions provided with the package.

##### Benchmarking process

1. Open the Tutorial/Benchmarking directory. Here, you will find everything you need: the labeled active and inactive datasets (GPCR_active_labeled.csv and GPCR_inactive_labeled.csv), the WT sequence and structure files, the amino acid properties file, and the pre‐trained model.
2. Load the data files (GPCR_active_labeled.csv and GPCR_inactive_labeled.csv) into your StabLyzeGraph GUI corresponding fields.
3. Tune the hyperparameters (use the default parameters first), then get the trained model and evaluate the metrics.
4. Check how well the model did by evaluating metrics like accuracy, F1‐score, AUC‐ROC, or correlation coefficients to see how StabLyzeGraph handles this dataset.
5. Record your results and re‐run until you find your preferred model.

##### Screening process

1. Start in the Tutorial/Screening directory, where you will find the trained model, WT protein information, and amino acid properties.
2. Load the data files (GPCR_active_labeled.csv and GPCR_inactive_labeled.csv) into your StabLyzeGraph GUI corresponding fields, just like the Benchmarking process.
3. By default, the same parameters will be used to screen mutants as for the trained model; also, you can fix the number of mutations per sequence (to generate mutants).
4. Use the best‐performing trained model to screen your mutations. The model will rank the mutants and show you the probability for each one, helping you easily identify the most promising candidates.

### 4.6 Graphical User Interface

**FIGURE 6:**
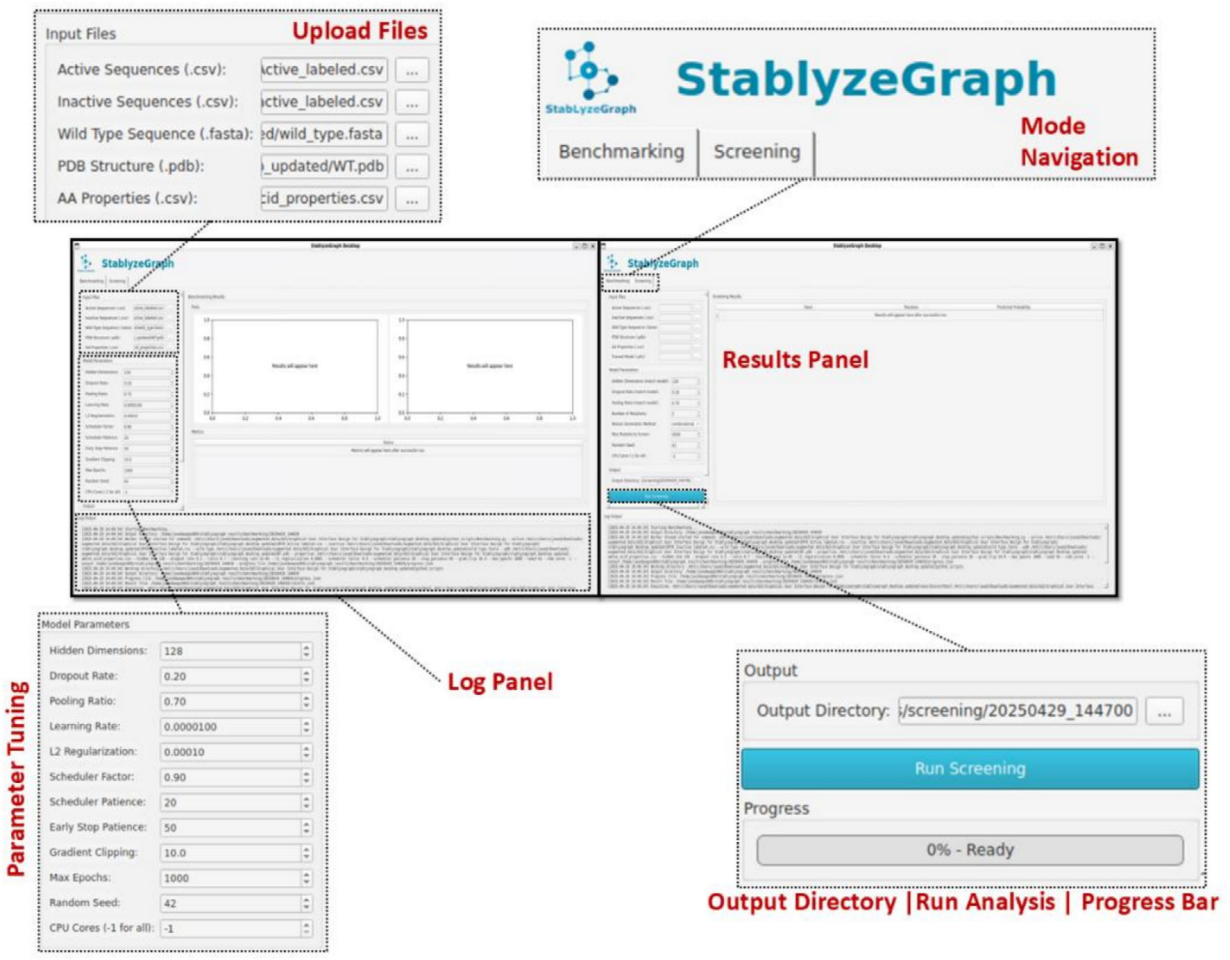
StabLyzeGraph interface: the user‐friendly interface and operational workflow of the StabLyzeGraph software. Benchmarking tab for initial input selection and Mutation analysis tab selecting models, number of mutations per sequence, and file uploads, along with the results panel.

The StabLyzeGraph GUI is developed with the PyQt5 package of Python and integrates both the benchmarking and mutation analysis (screening) modules. It provides an intuitive, user‐friendly workflow, making it accessible and streamlined for researchers, particularly biologists from various disciplines (Willman, 2021). This GUI framework has two main tabs to switch between the modules. In the benchmarking module, users must upload protein sequence (mutants) and amino acid properties for feature generation as CSV files, the WT fasta sequence, and the WT protein structure file in PDB format. Model performance metrics can be analyzed after the completion of benchmarking in the same window. Also, in the mutation analysis tab, users must upload the same files as benchmarking mode along with the trained model; then analysis will start with the run button, and results are provided as a CSV file at process completion (Figure 6).

## AUTHOR CONTRIBUTIONS

**Muhammad Waqas:** Writing – original draft; methodology; software; investigation; data curation. **Benito Natale:** Investigation; methodology; software; writing – original draft; validation. **Michele Roggia:** Investigation; methodology; writing – original draft; software; validation; formal analysis. **Prasenjit Prasad Saha:** Writing – review and editing. **Mauro Mileni:** Supervision; writing – review and editing; visualization. **Sandro Cosconati:** Conceptualization; writing – original draft; writing – review and editing; project administration; supervision; software; funding acquisition; formal analysis; resources.

## CONFLICT OF INTEREST STATEMENT

The authors declare no conflicts of interest.

## Supporting information


**Data S1.** Supporting Information.

**Data S2.** Supporting Information.

## Data Availability

The data that support the findings of this study are available from the corresponding author upon reasonable request. StabLyzeGraph is freely available on GitHub (https://github.com/cosconatilab/StabLyzeGraph) as an open‐source tool.
