# Supplementary material for: StabLyzeGraph: High‐throughput screening of combinatorial mutations using graph neural networks
Source: Protein Sci. 2026 Mar 7;35(4):e70534. doi: 10.1002/pro.70534 (PMC12967658; doi:10.1002/pro.70534)
Supplement: Supplementary file 1 — Data S1. Supporting Information. [file PRO-35-e70534-s002.docx]

| **DATASETS** | **Protein Length** | **Actives** | **Inactives** | **Optimal Threshold** | **Hidden** | **Dropout** | **Ratio** | **LR** | **L2** | **Sc. Factor** | **Sc. Patience** | **St. Patience** | **Max. Epochs** |
| --- | --- | --- | --- | --- | --- | --- | --- | --- | --- | --- | --- | --- | --- |
| **Dataset 1** | 164 | 75 | 215 | 0.989013 | 128 | 0.2 | 0.75 | 0.00001 | 0.0001 | 0.1 | 20 | 50 | 1000 |
| **Dataset 2** | 354 | 47 | 302 | 0.740371 | 128 | 0.2 | 0.75 | 0.00001 | 0.0001 | 0.9 | 20 | 50 | 300 |
| **Dataset 3** | 130 | 13 | 52 | 0.878181 | 128 | 0.2 | 0.75 | 0.00001 | 0.0001 | 0.9 | 20 | 50 | 700 |
| **Dataset 4** | 302 | 76 | 47 | 0.626796 | 128 | 0.2 | 0.75 | 0.00001 | 0.0001 | 0.9 | 20 | 50 | 120 |
| **Dataset 5** | 254 | 66 | 89 | 0.726007 | 128 | 0.2 | 0.75 | 0.00001 | 0.0001 | 0.9 | 10 | 50 | 500 |
| **Dataset 6** | 448 | 239 | 579 | 0.891767 | 128 | 0.2 | 0.75 | 0.00001 | 0.0001 | 0.5 | 20 | 50 | 1000 |
| **Dataset 7** | 148 | 31 | 90 | 0.896474 | 128 | 0.2 | 0.75 | 0.00001 | 0.0001 | 0.9 | 20 | 50 | 800 |
| **Dataset 8** | 153 | 74 | 14 | 0.736546 | 128 | 0.2 | 0.75 | 0.00001 | 0.0001 | 0.1 | 10 | 50 | 850 |
| **Dataset 12** | 155 | 42 | 35 | 0.775682 | 128 | 0.2 | 0.75 | 0.00001 | 0.0001 | 0.9 | 20 | 50 | 500 |
| **Dataset 16** | 231 | 59 | 498 | 0.668278 | 128 | 0.2 | 0.75 | 0.00001 | 0.0001 | 0.9 | 10 | 50 | 1000 |
| **Dataset 17** | 268 | 27 | 19 | 0.759841 | 128 | 0.2 | 0.75 | 0.00001 | 0.0001 | 0.9 | 20 | 50 | 1000 |
| **Dataset 18** | 537 | 12 | 80 | 0.817507 | 128 | 0.2 | 0.75 | 0.00001 | 0.0001 | 0.9 | 10 | 50 | 500 |
| **Dataset 19** | 170 | 24 | 19 | 0.639839 | 128 | 0.2 | 0.75 | 0.00001 | 0.0001 | 0.9 | 10 | 50 | 800 |
| **Dataset 21** | 159 | 20 | 75 | 0.946166 | 128 | 0.2 | 0.75 | 0.00001 | 0.0001 | 0.1 | 10 | 50 | 800 |
| **Dataset 23** | 147 | 24 | 38 | 0.645289 | 128 | 0.2 | 0.75 | 0.00001 | 0.0001 | 0.9 | 10 | 50 | 600 |
| **Dataset P00644** | 231 | 413 | 69 | 0.476931 | 128 | 0.2 | 0.75 | 0.00001 | 0.0001 | 0.9 | 10 | 50 | 1000 |
| **Dataset P00648** | 157 | 136 | 17 | 0.636627 | 128 | 0.2 | 0.75 | 0.00001 | 0.0001 | 0.9 | 10 | 50 | 600 |
| **Dataset P00720** | 164 | 67 | 31 | 0.567639 | 128 | 0.2 | 0.75 | 0.00001 | 0.0001 | 0.9 | 20 | 50 | 250 |
| **Dataset 5H2C** | 458 | 19 | 14 | 0.426955 | 128 | 0.2 | 0.75 | 0.00001 | 0.0001 | 0.9 | 20 | 50 | 85 |
| **Dataset ADGRL1** | 1515 | 51 | 15 | 0.491083 | 128 | 0.2 | 0.75 | 0.00001 | 0.0001 | 0.9 | 20 | 50 | 100 |

**Table 1: Model Evaluation and hyperparameters** for all datasets.


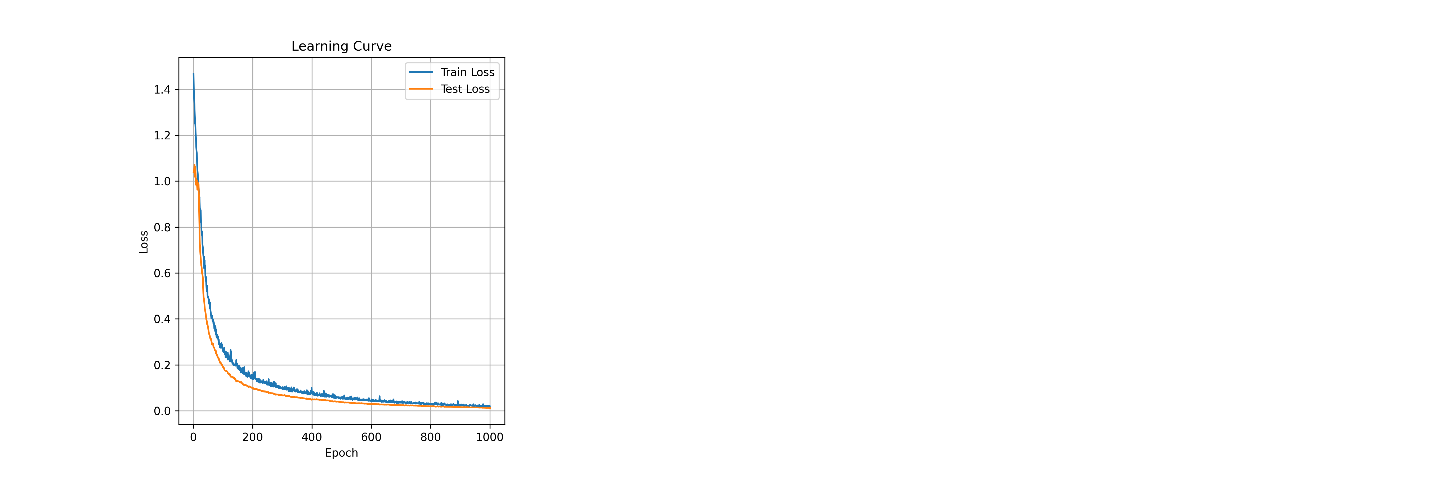

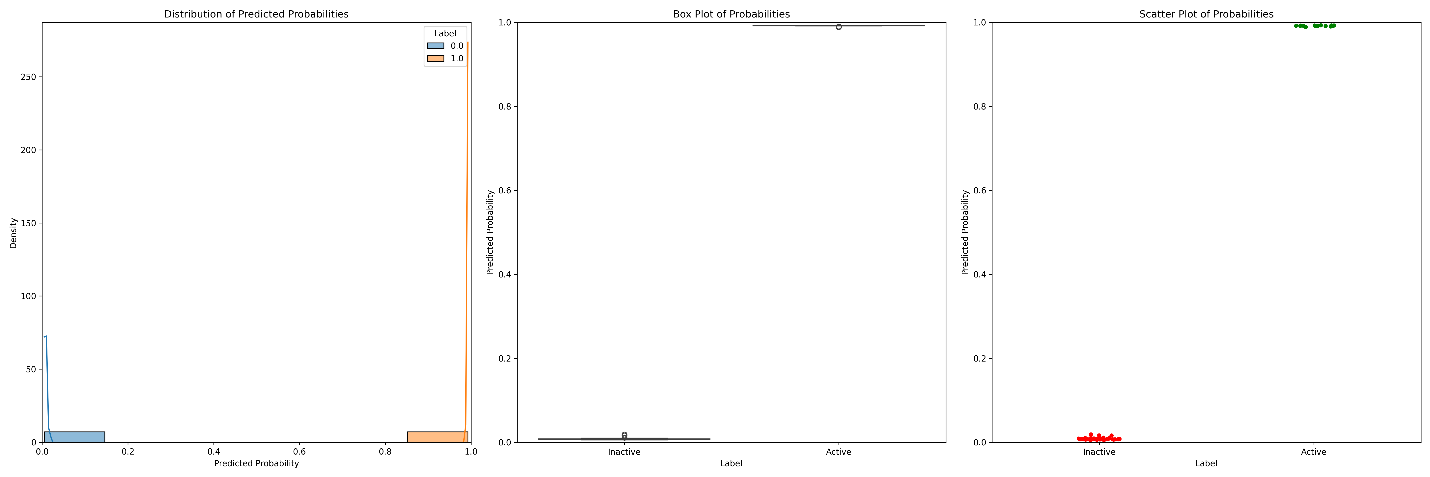


**Figure 1: Benchmarking Dataset 1: Train and Test loss,** train loss is displayed as blue curve and test loss is depicted by orange curve. **Distribution of Probabilities,** as a histogram can be visualized having Blue (Inactive) and Orange (Active) Bars. **Box Plot of Probabilities,** determining the clear separation between active/inactive class and having a good balance between the prediction, and **Scatter Plot of Probabilities,** shows the confidence in the probabilities for both active (green) and inactive (orange) dots.


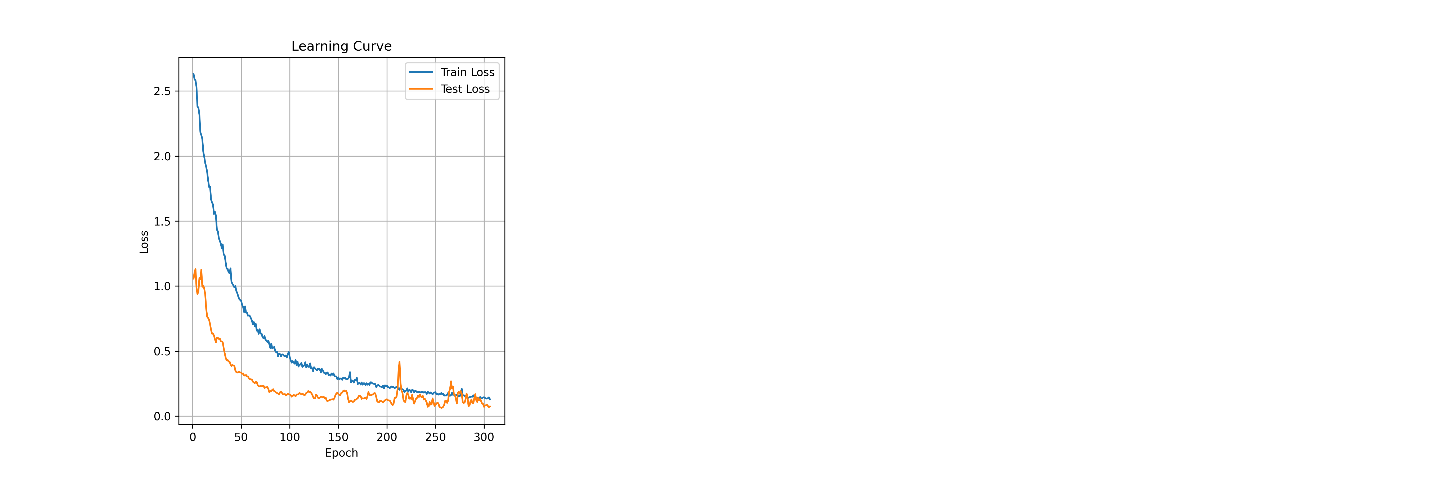

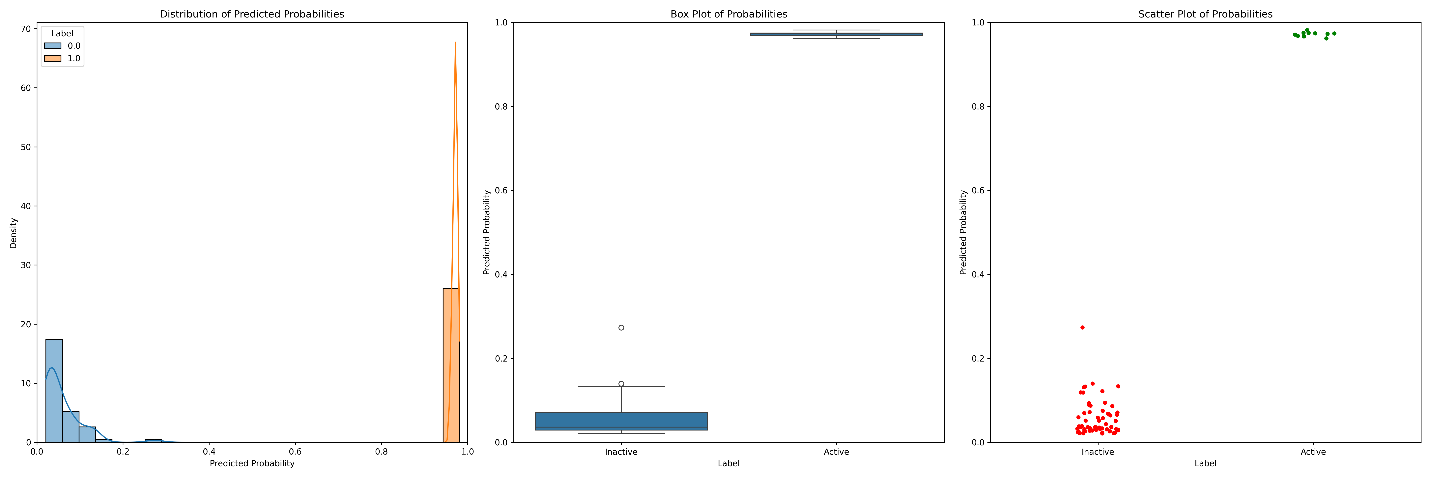


**Figure 2: Benchmarking Dataset 2: Train and Test loss,** train loss is displayed as blue curve and test loss is depicted by orange curve. **Distribution of Probabilities,** as a histogram can be visualized having Blue (Inactive) and Orange (Active) Bars. **Box Plot of Probabilities,** determining the clear separation between active/inactive class and having a good balance between the prediction, and **Scatter Plot of Probabilities,** shows the confidence in the probabilities for both active (green) and inactive (orange) dots.


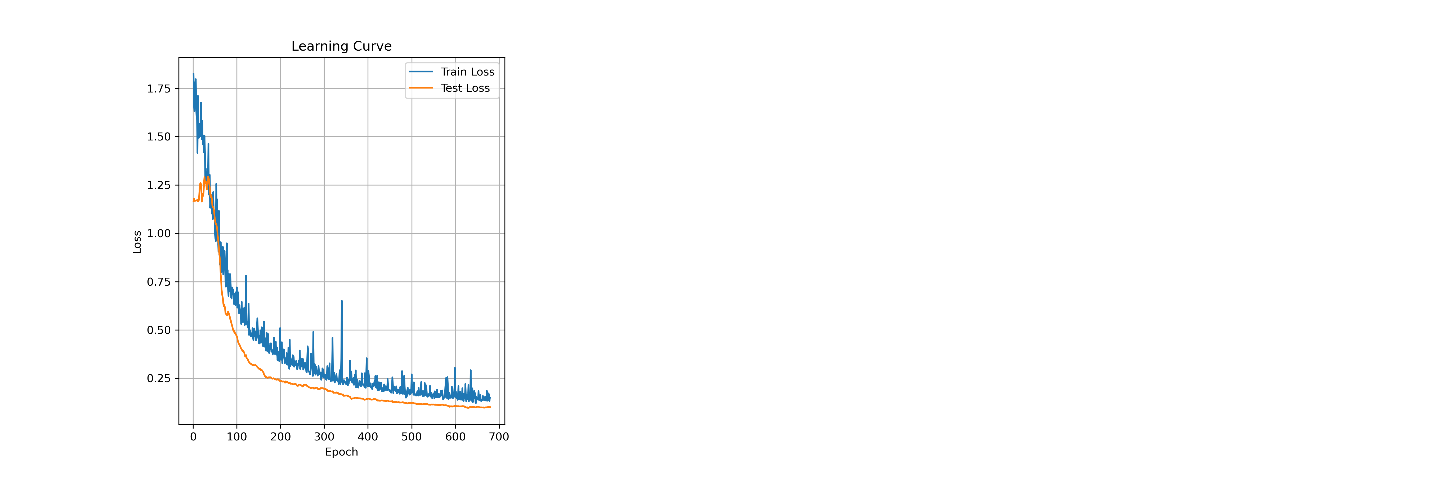

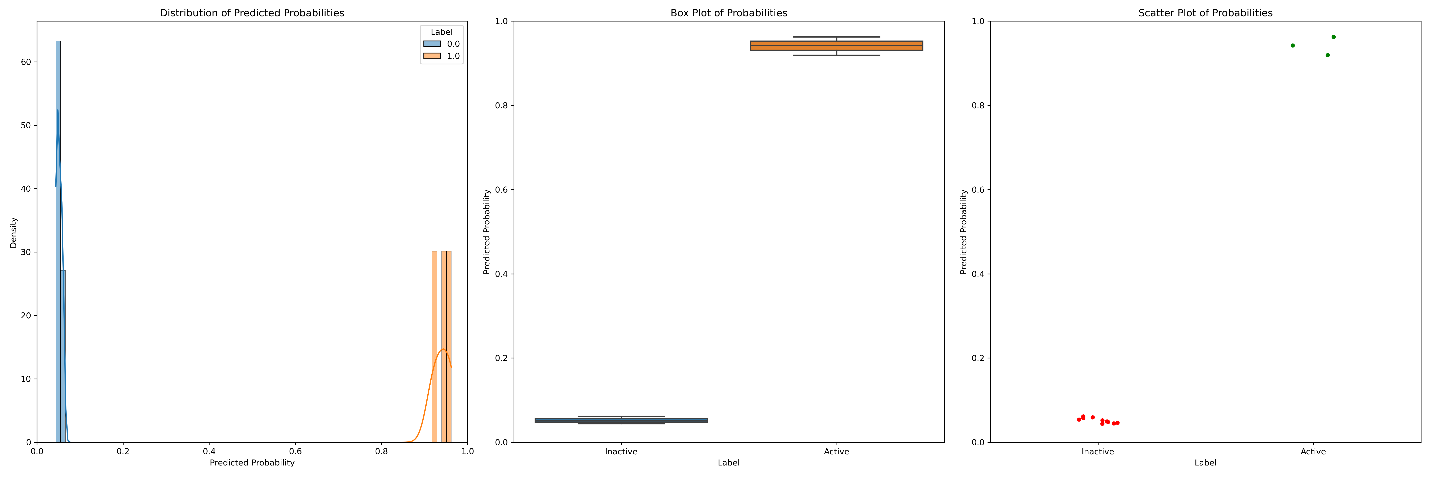


**Figure 3: Benchmarking Dataset 3: Train and Test loss,** train loss is displayed as blue curve and test loss is depicted by orange curve. **Distribution of Probabilities,** as a histogram can be visualized having Blue (Inactive) and Orange (Active) Bars. **Box Plot of Probabilities,** determining the clear separation between active/inactive class and having a good balance between the prediction, and **Scatter Plot of Probabilities,** shows the confidence in the probabilities for both active (green) and inactive (orange) dots.


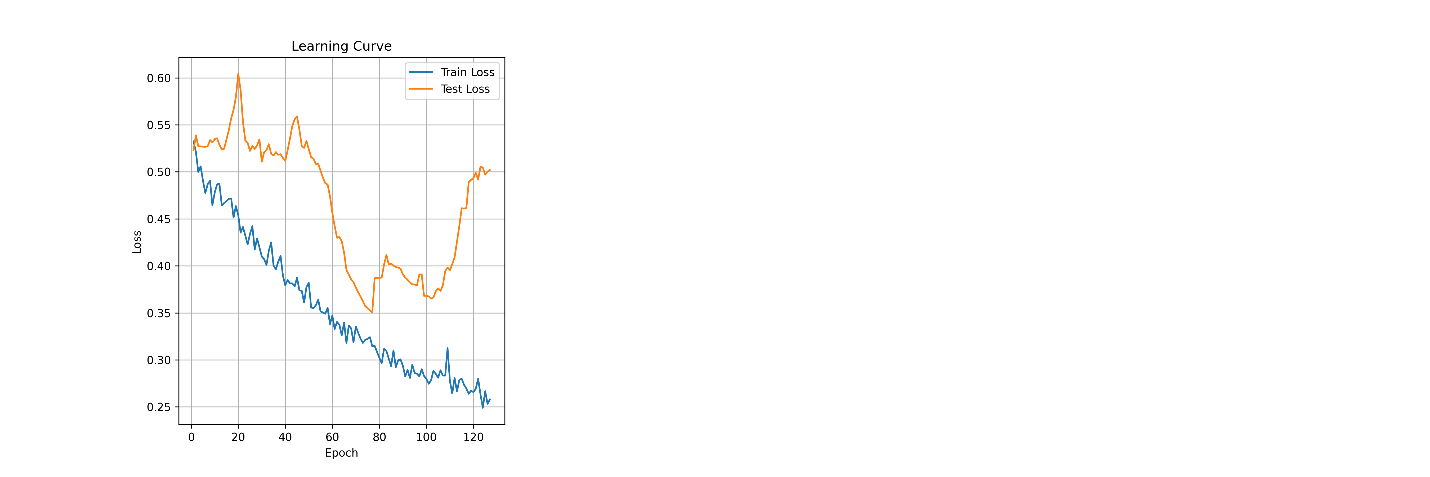

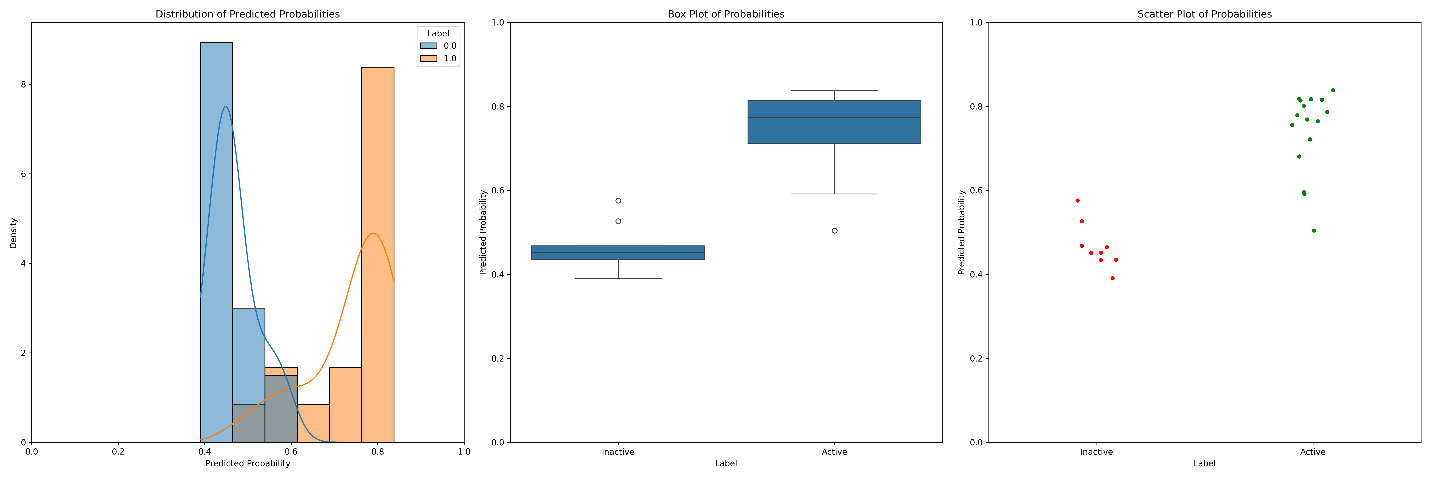


**Figure 4: Benchmarking Dataset 4: Train and Test loss,** train loss is displayed as blue curve and test loss is depicted by orange curve. **Distribution of Probabilities,** as a histogram can be visualized having Blue (Inactive) and Orange (Active) Bars. **Box Plot of Probabilities,** determining the clear separation between active/inactive class and having a good balance between the prediction, and **Scatter Plot of Probabilities,** shows the confidence in the probabilities for both active (green) and inactive (orange) dots.


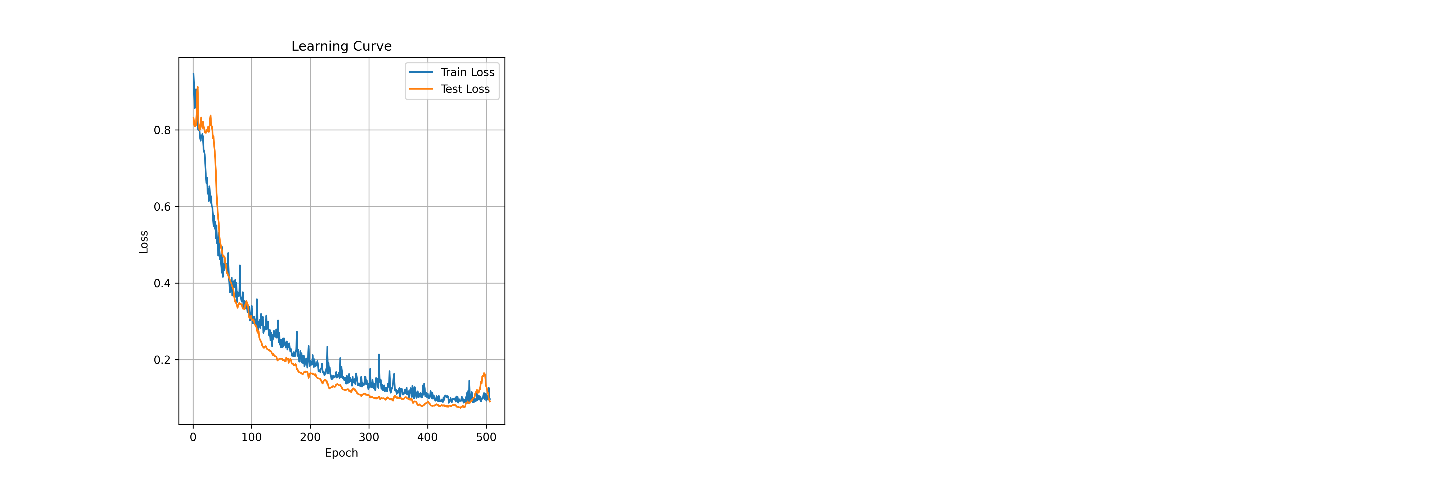

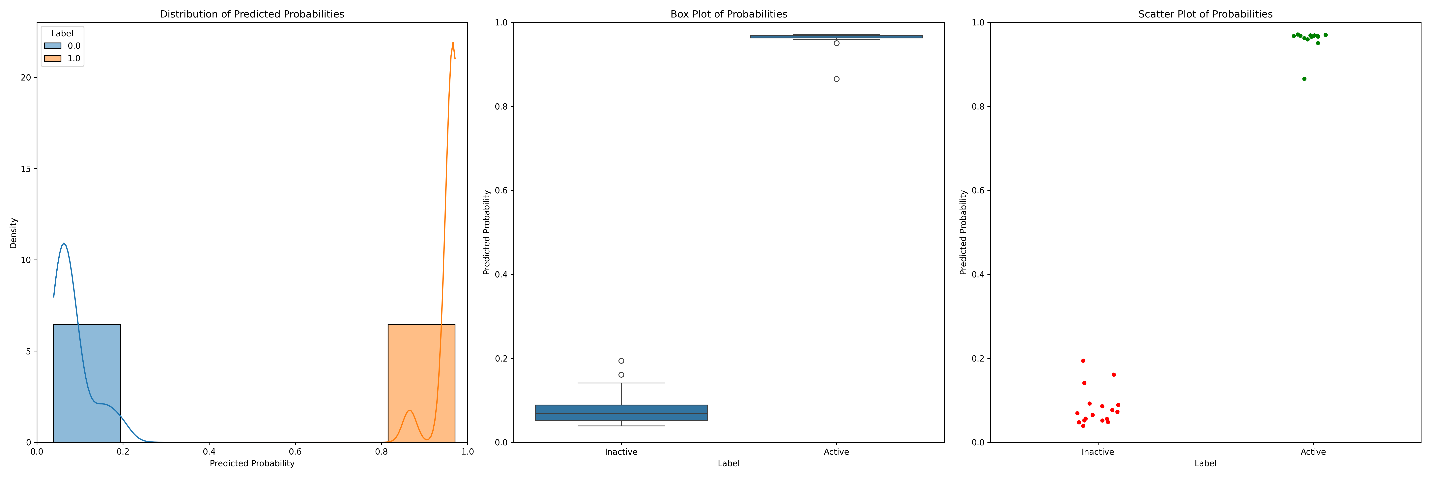


**Figure 5: Benchmarking Dataset 5: Train and Test loss,** train loss is displayed as blue curve and test loss is depicted by orange curve. **Distribution of Probabilities,** as a histogram can be visualized having Blue (Inactive) and Orange (Active) Bars. **Box Plot of Probabilities,** determining the clear separation between active/inactive class and having a good balance between the prediction, and **Scatter Plot of Probabilities,** shows the confidence in the probabilities for both active (green) and inactive (orange) dots.


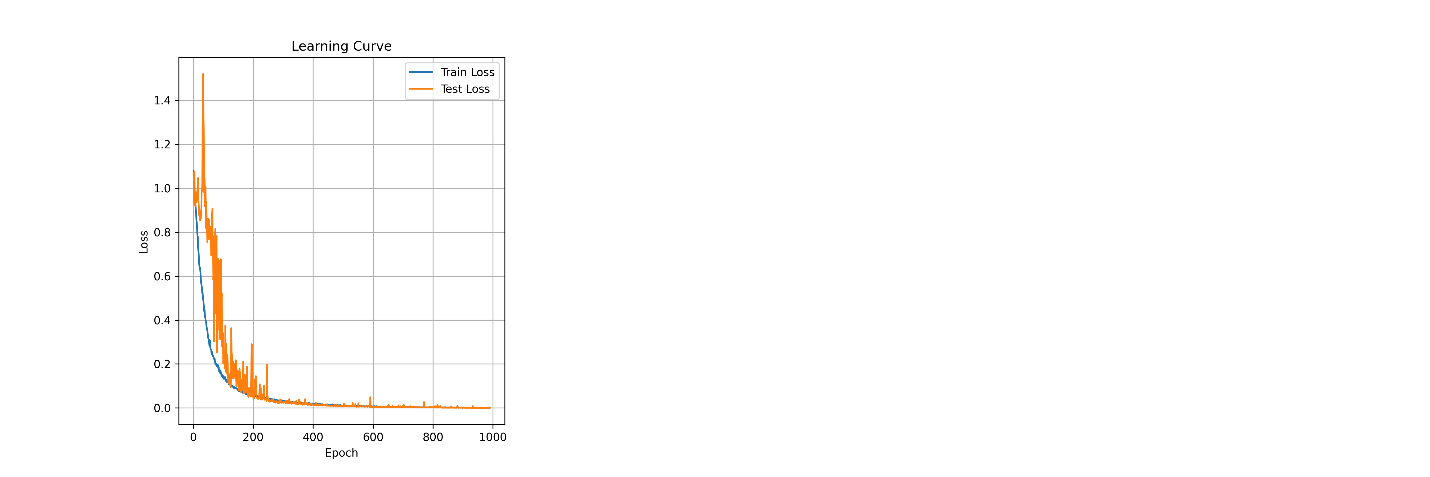

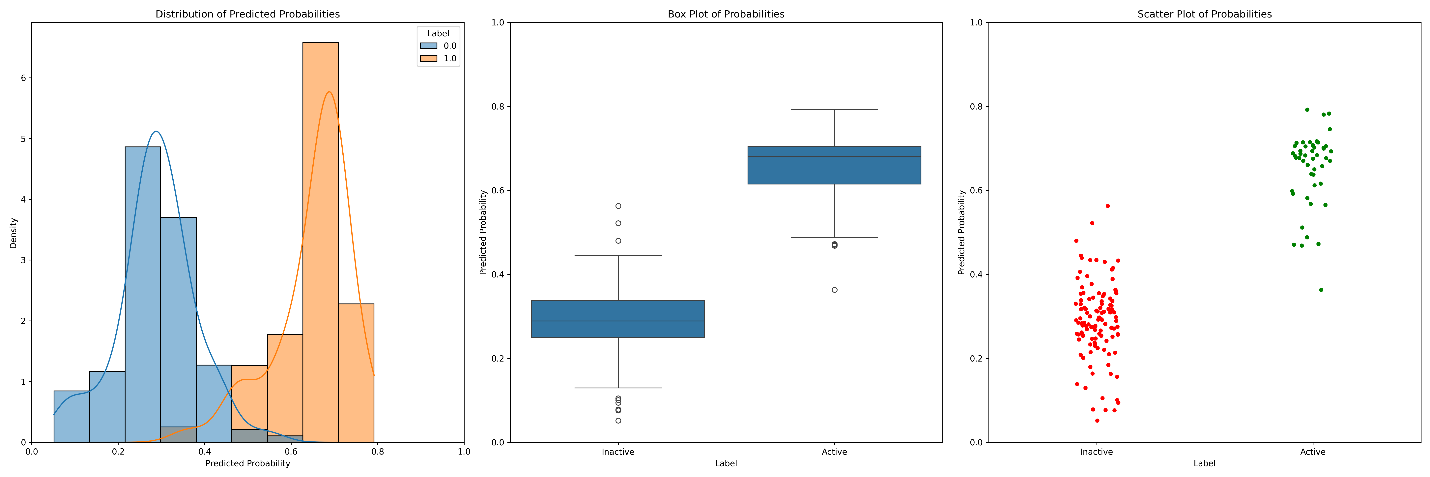


**Figure 6: Benchmarking Dataset 6: Train and Test loss,** train loss is displayed as blue curve and test loss is depicted by orange curve. **Distribution of Probabilities,** as a histogram can be visualized having Blue (Inactive) and Orange (Active) Bars. **Box Plot of Probabilities,** determining the clear separation between active/inactive class and having a good balance between the prediction, and **Scatter Plot of Probabilities,** shows the confidence in the probabilities for both active (green) and inactive (orange) dots.


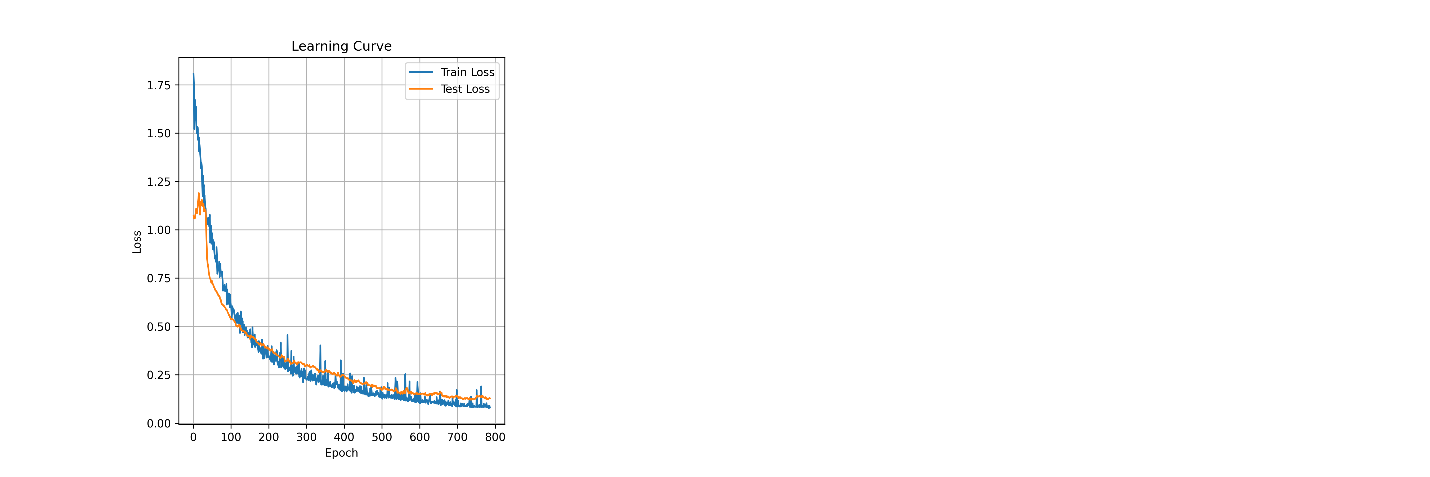

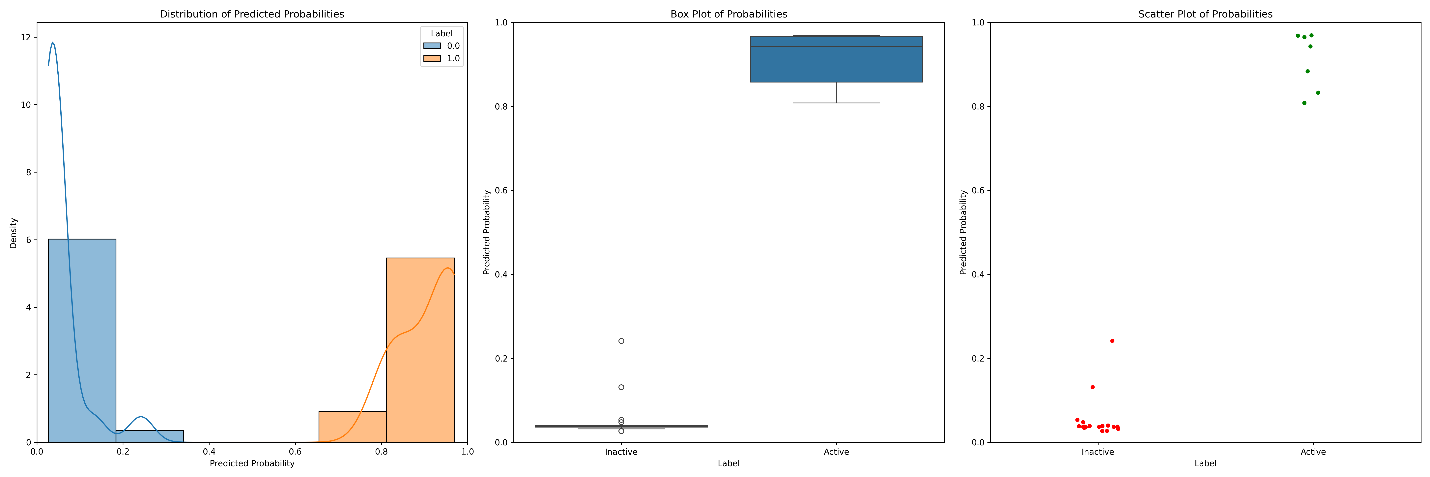


**Figure 7: Benchmarking Dataset 7: Train and Test loss,** train loss is displayed as blue curve and test loss is depicted by orange curve. **Distribution of Probabilities,** as a histogram can be visualized having Blue (Inactive) and Orange (Active) Bars. **Box Plot of Probabilities,** determining the clear separation between active/inactive class and having a good balance between the prediction, and **Scatter Plot of Probabilities,** shows the confidence in the probabilities for both active (green) and inactive (orange) dots.


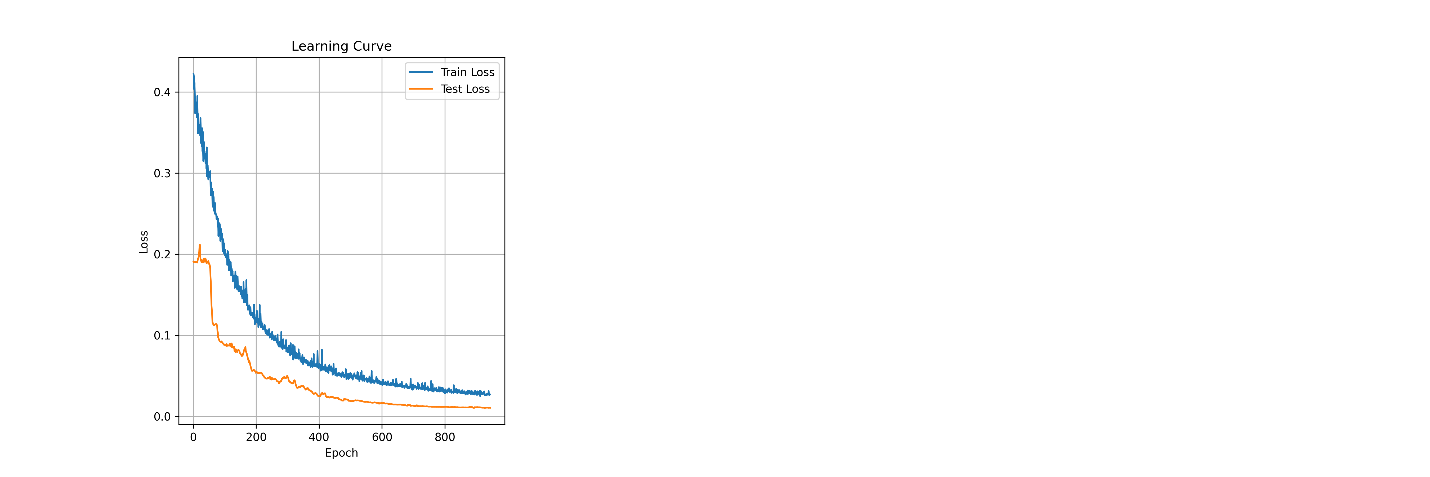

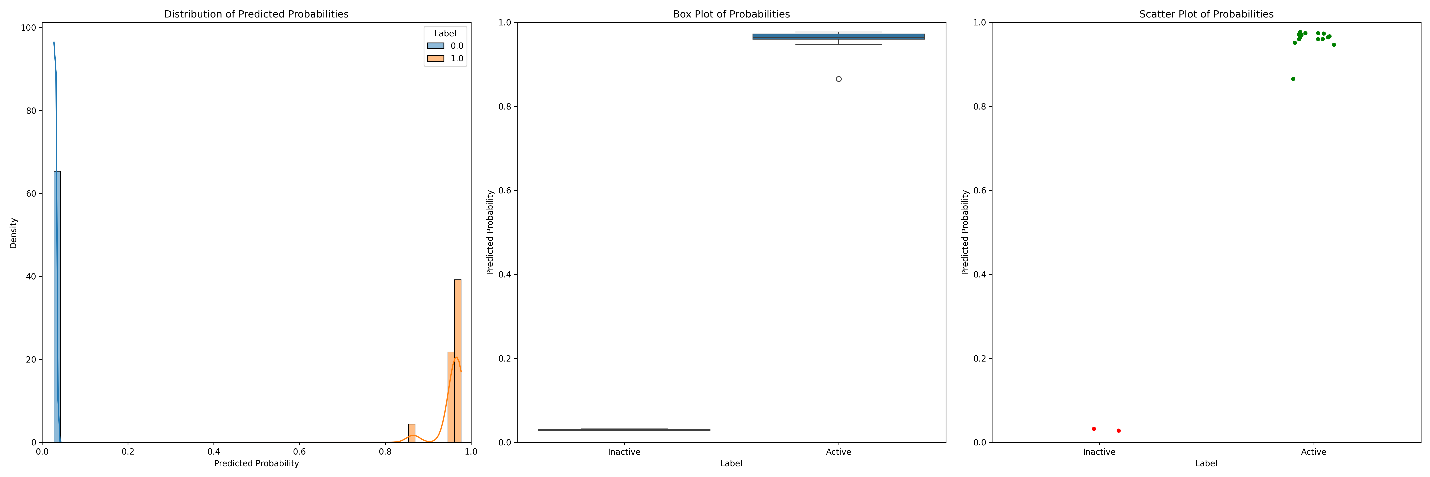


**Figure 8: Benchmarking Dataset 8: Train and Test loss,** train loss is displayed as blue curve and test loss is depicted by orange curve. **Distribution of Probabilities,** as a histogram can be visualized having Blue (Inactive) and Orange (Active) Bars. **Box Plot of Probabilities,** determining the clear separation between active/inactive class and having a good balance between the prediction, and **Scatter Plot of Probabilities,** shows the confidence in the probabilities for both active (green) and inactive (orange) dots.


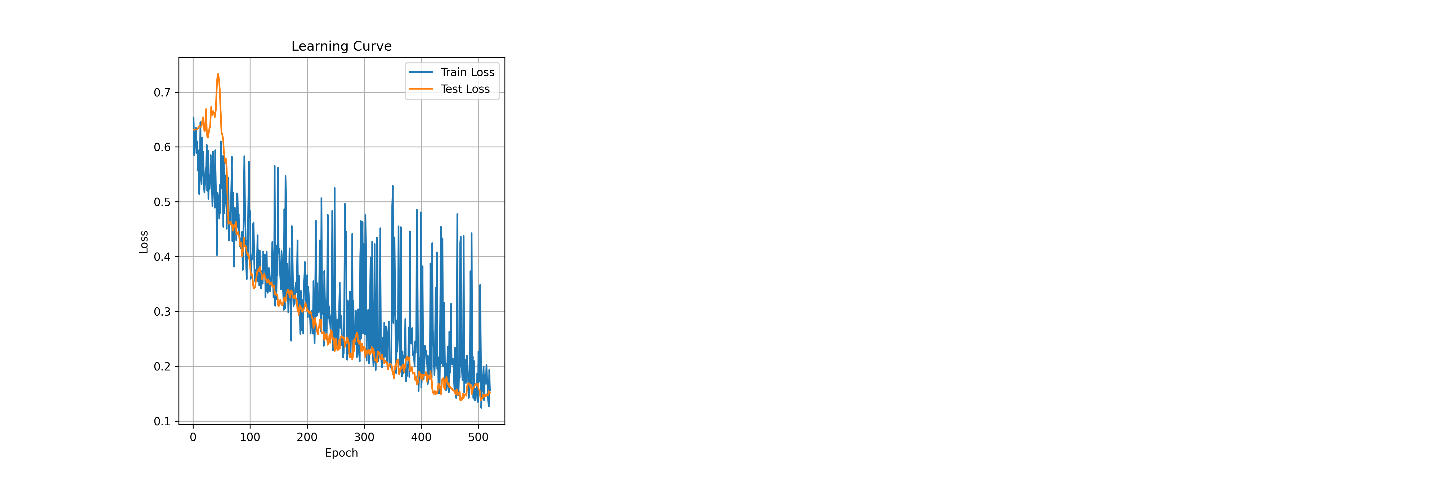

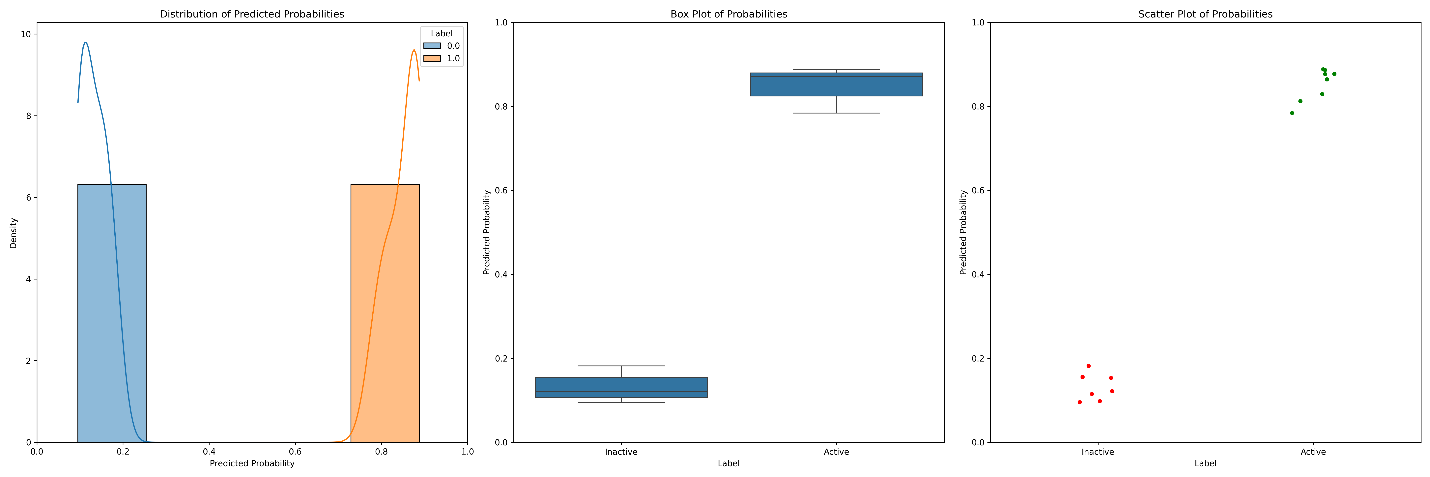


**Figure 9: Benchmarking Dataset 12: Train and Test loss,** train loss is displayed as blue curve and test loss is depicted by orange curve. **Distribution of Probabilities,** as a histogram can be visualized having Blue (Inactive) and Orange (Active) Bars. **Box Plot of Probabilities,** determining the clear separation between active/inactive class and having a good balance between the prediction, and **Scatter Plot of Probabilities,** shows the confidence in the probabilities for both active (green) and inactive (orange) dots.


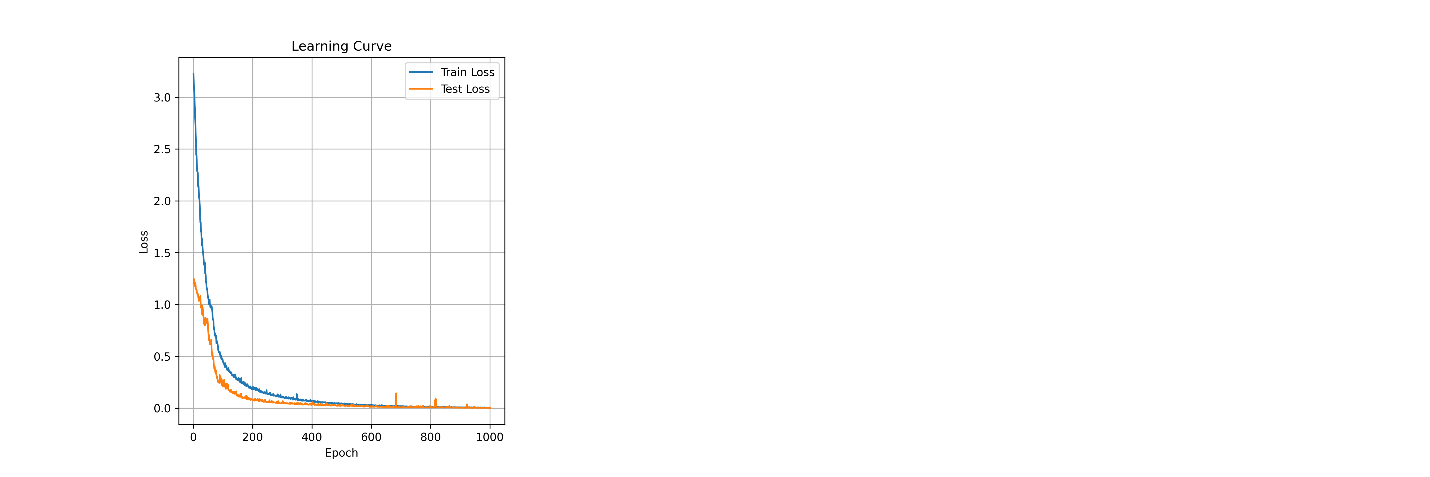

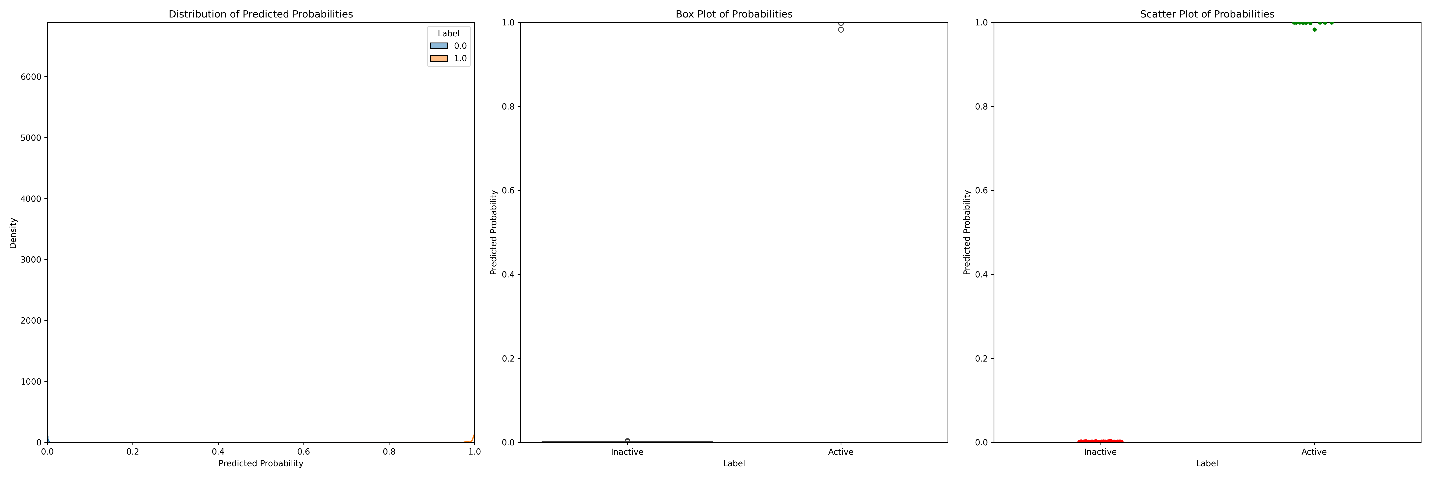


**Figure 10: Benchmarking Dataset 16: Train and Test loss,** train loss is displayed as blue curve and test loss is depicted by orange curve. **Distribution of Probabilities,** as a histogram can be visualized having Blue (Inactive) and Orange (Active) Bars. **Box Plot of Probabilities,** determining the clear separation between active/inactive class and having a good balance between the prediction, and **Scatter Plot of Probabilities,** shows the confidence in the probabilities for both active (green) and inactive (orange) dots.


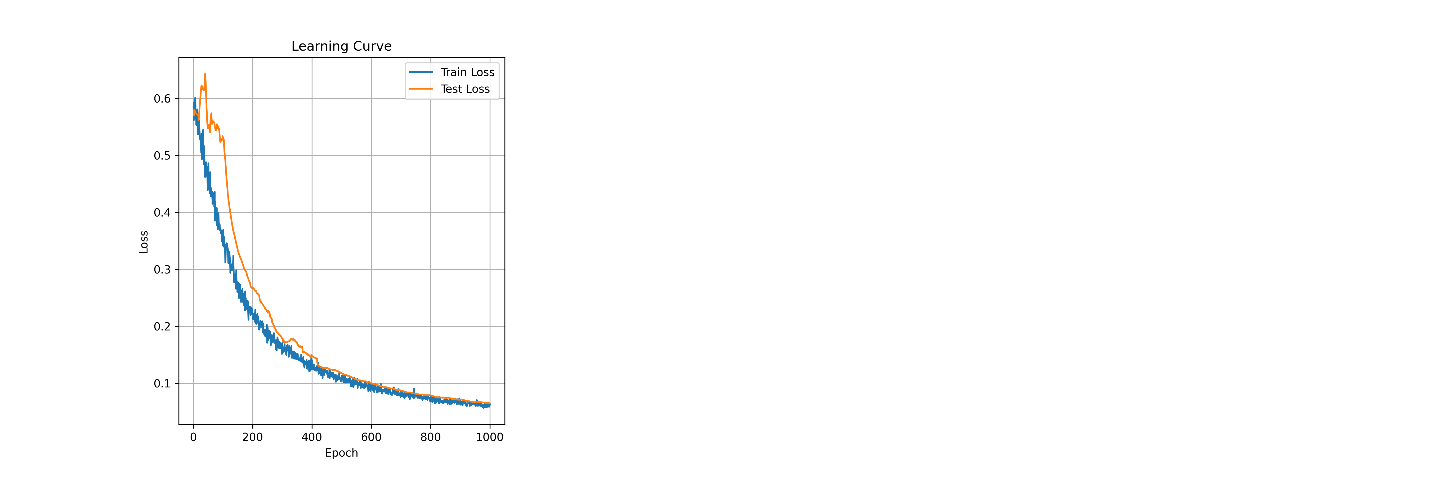

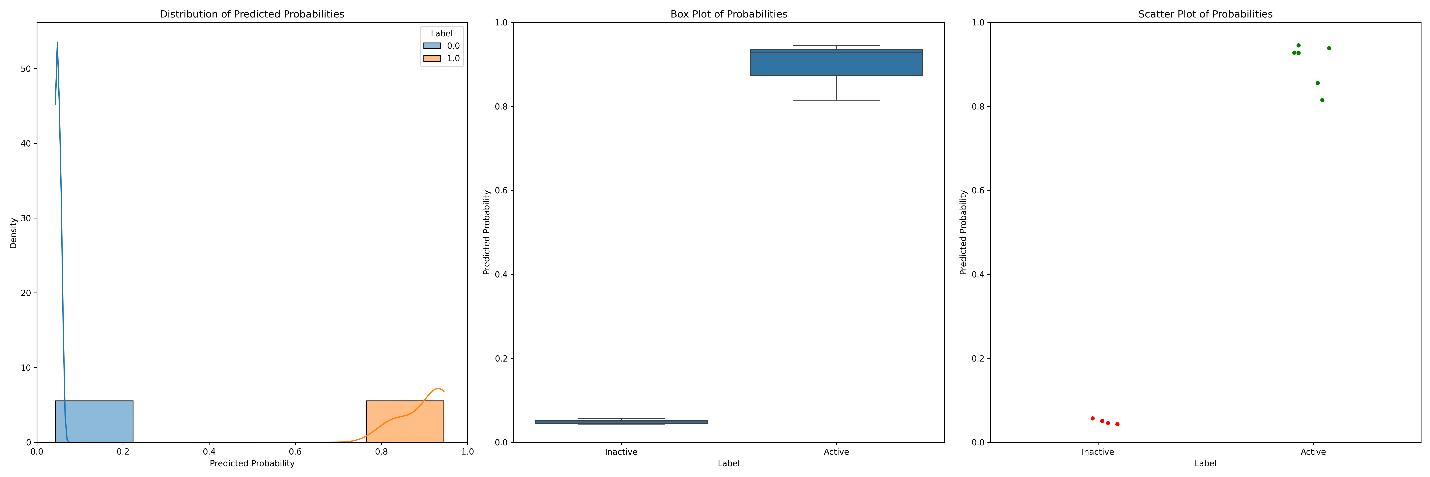


**Figure 11: Benchmarking Dataset 17: Train and Test loss,** train loss is displayed as blue curve and test loss is depicted by orange curve. **Distribution of Probabilities,** as a histogram can be visualized having Blue (Inactive) and Orange (Active) Bars. **Box Plot of Probabilities,** determining the clear separation between active/inactive class and having a good balance between the prediction, and **Scatter Plot of Probabilities,** shows the confidence in the probabilities for both active (green) and inactive (orange) dots.


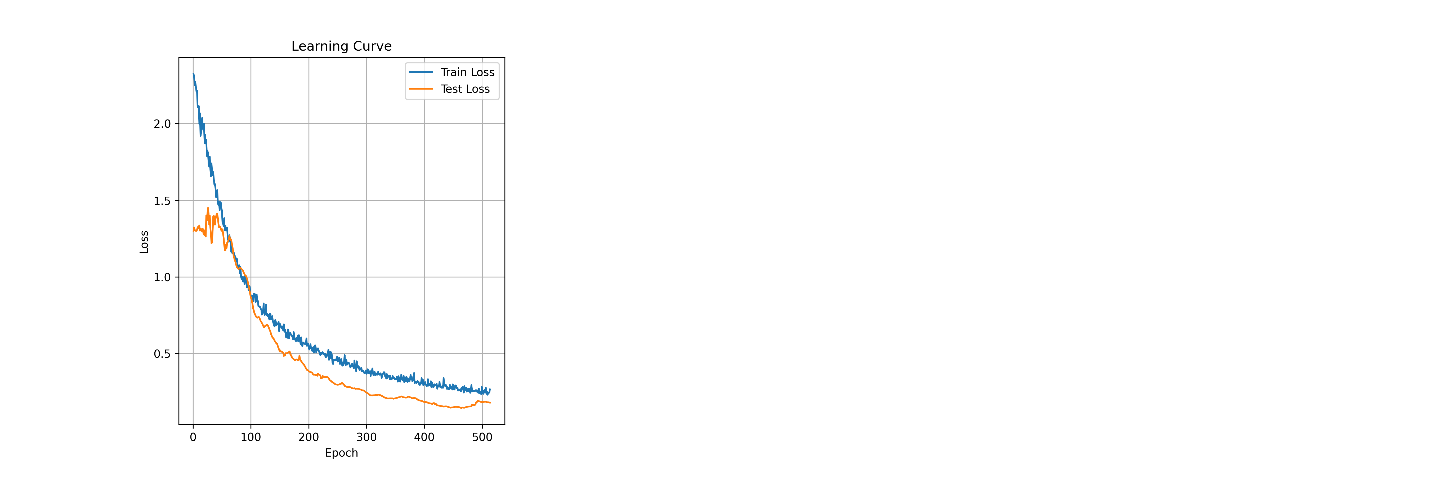

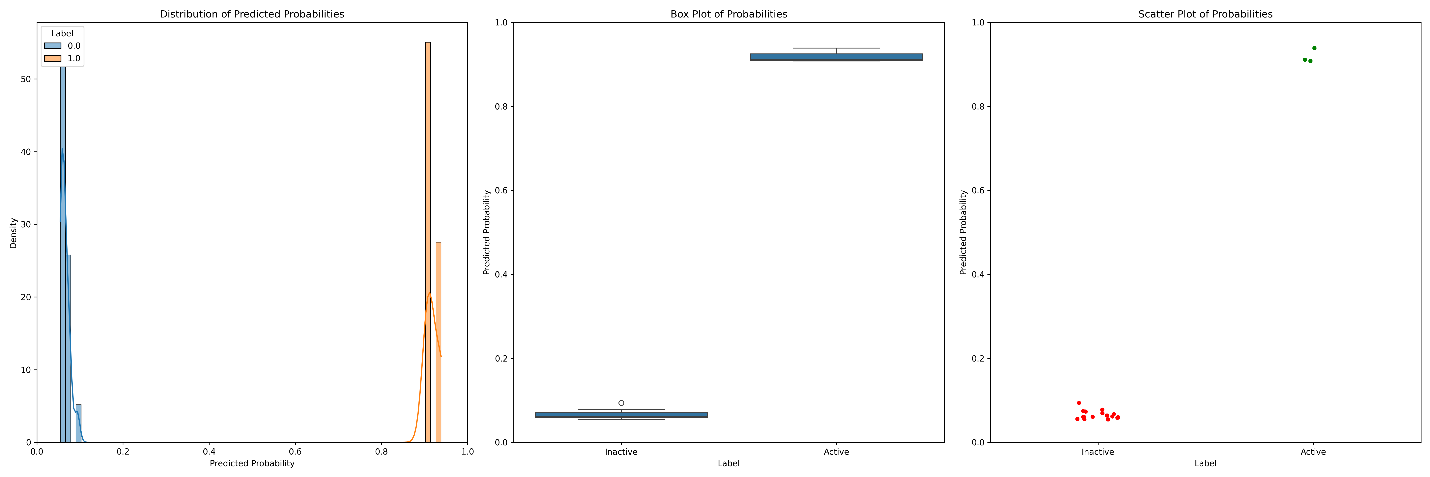


**Figure 12: Benchmarking Dataset 18: Train and Test loss,** train loss is displayed as blue curve and test loss is depicted by orange curve. **Distribution of Probabilities,** as a histogram can be visualized having Blue (Inactive) and Orange (Active) Bars. **Box Plot of Probabilities,** determining the clear separation between active/inactive class and having a good balance between the prediction, and **Scatter Plot of Probabilities,** shows the confidence in the probabilities for both active (green) and inactive (orange) dots.


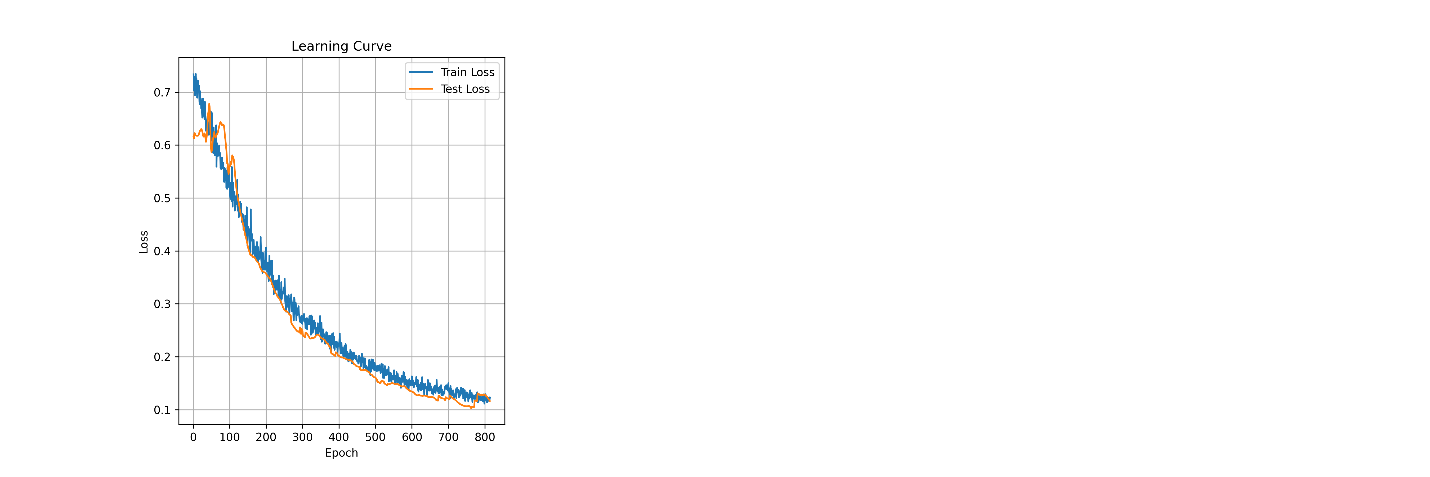

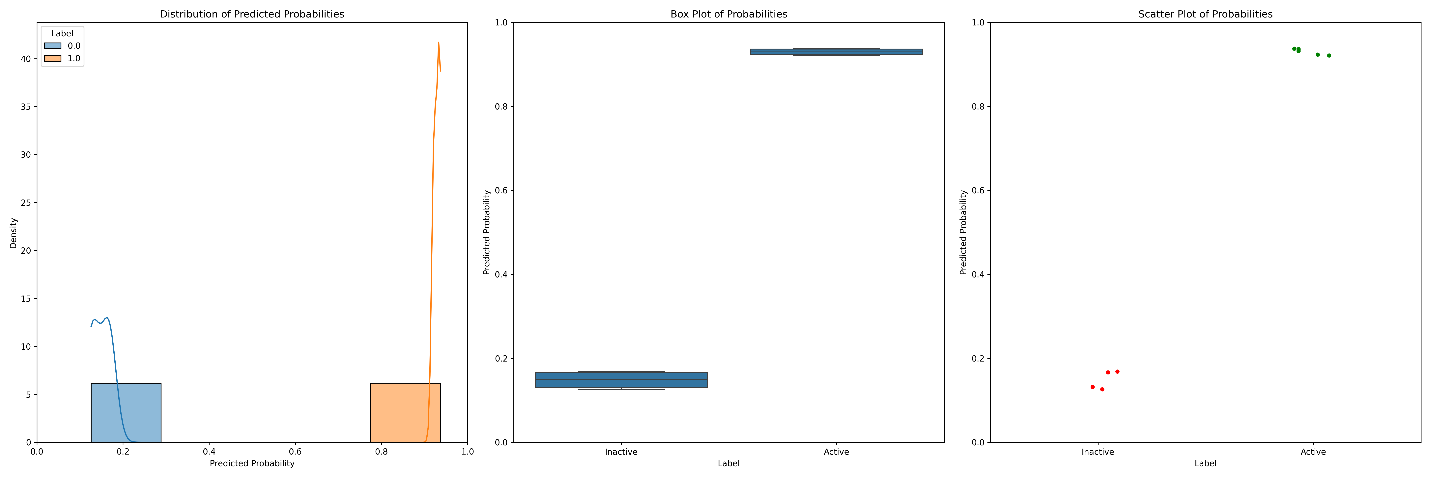


**Figure 13: Benchmarking Dataset 19: Train and Test loss,** train loss is displayed as blue curve and test loss is depicted by orange curve. **Distribution of Probabilities,** as a histogram can be visualized having Blue (Inactive) and Orange (Active) Bars. **Box Plot of Probabilities,** determining the clear separation between active/inactive class and having a good balance between the prediction, and **Scatter Plot of Probabilities,** shows the confidence in the probabilities for both active (green) and inactive (orange) dots.


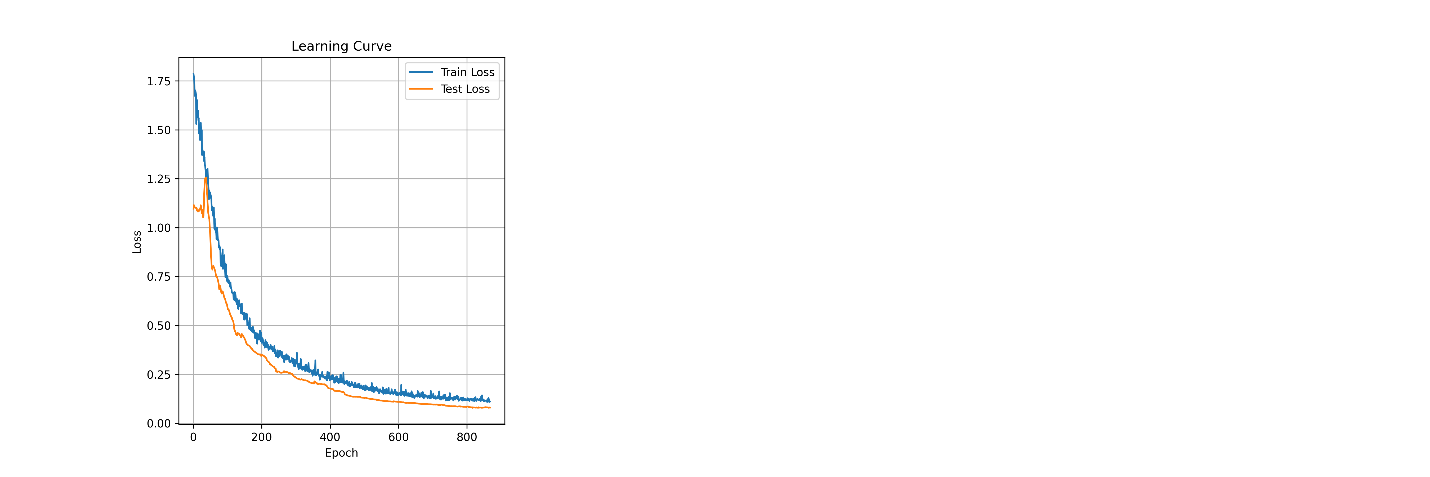

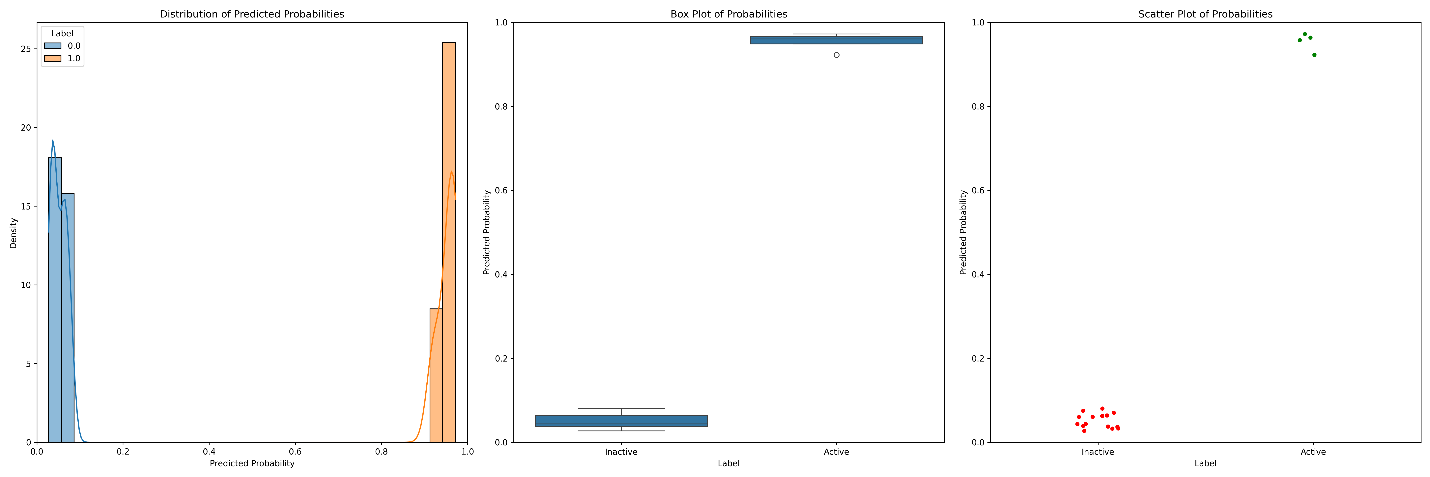


**Figure 14: Benchmarking Dataset 21: Train and Test loss,** train loss is displayed as blue curve and test loss is depicted by orange curve. **Distribution of Probabilities,** as a histogram can be visualized having Blue (Inactive) and Orange (Active) Bars. **Box Plot of Probabilities,** determining the clear separation between active/inactive class and having a good balance between the prediction, and **Scatter Plot of Probabilities,** shows the confidence in the probabilities for both active (green) and inactive (orange) dots.


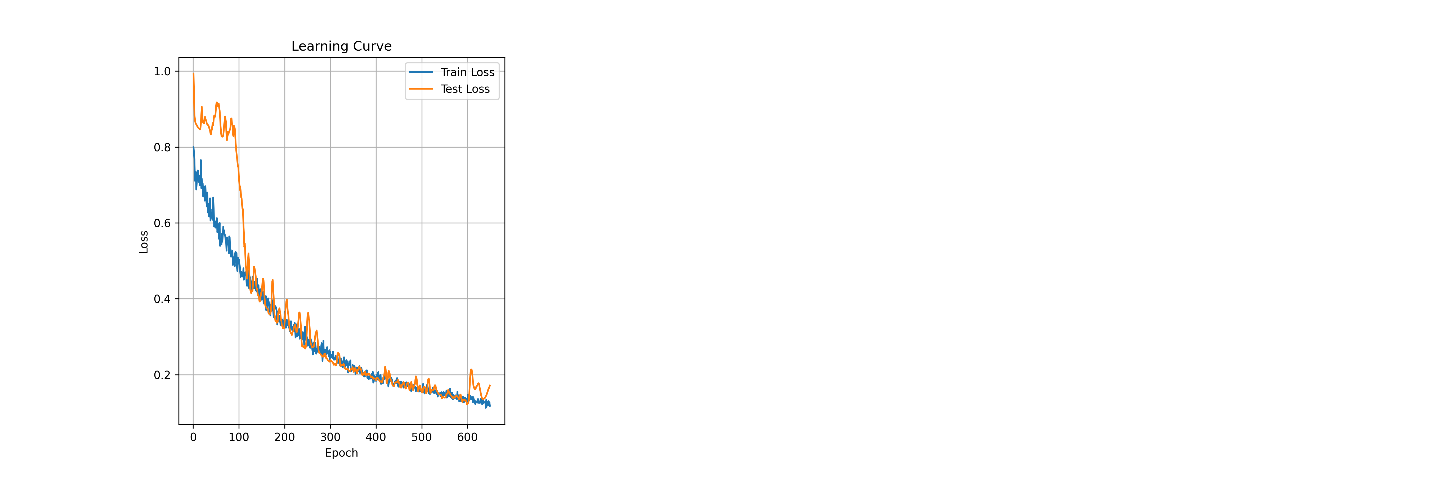

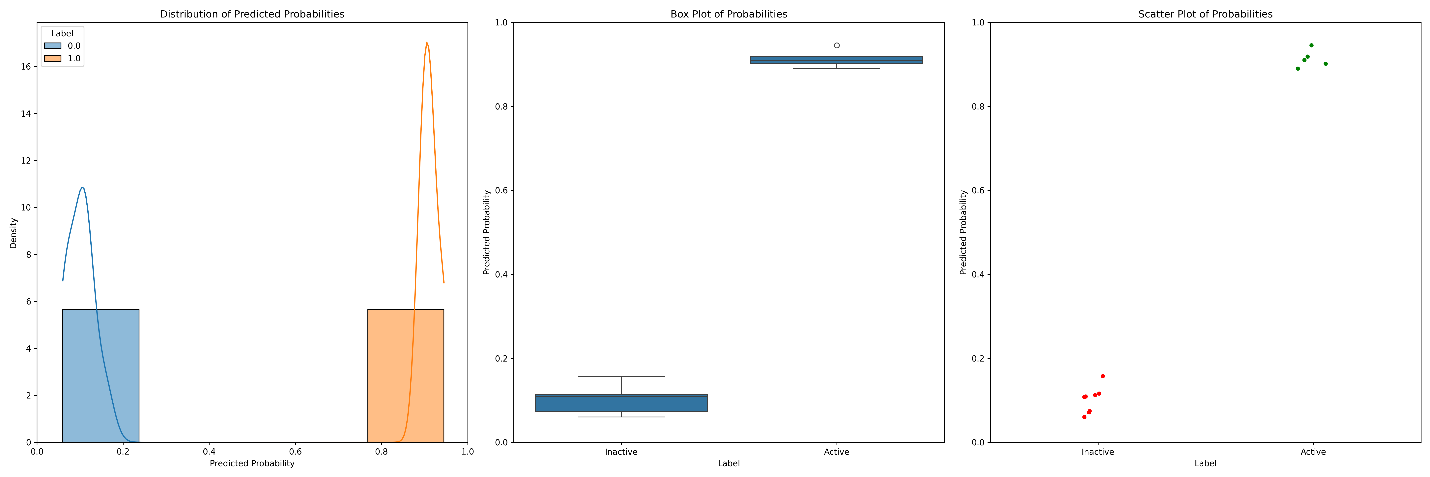


**Figure 15: Benchmarking Dataset 23: Train and Test loss,** train loss is displayed as blue curve and test loss is depicted by orange curve. **Distribution of Probabilities,** as a histogram can be visualized having Blue (Inactive) and Orange (Active) Bars. **Box Plot of Probabilities,** determining the clear separation between active/inactive class and having a good balance between the prediction, and **Scatter Plot of Probabilities,** shows the confidence in the probabilities for both active (green) and inactive (orange) dots.


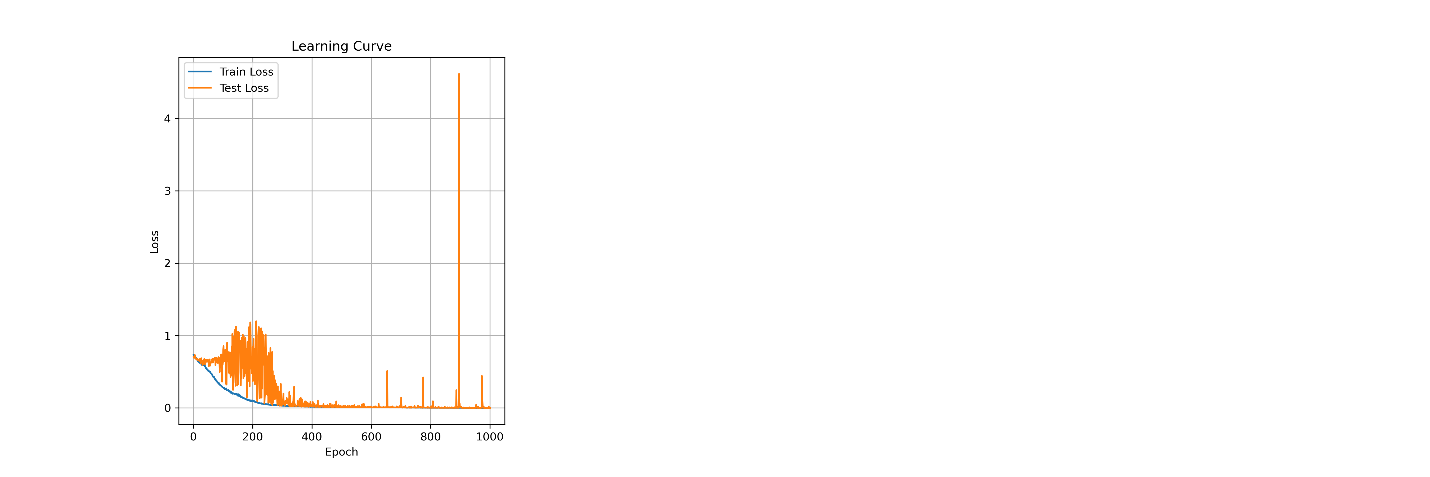

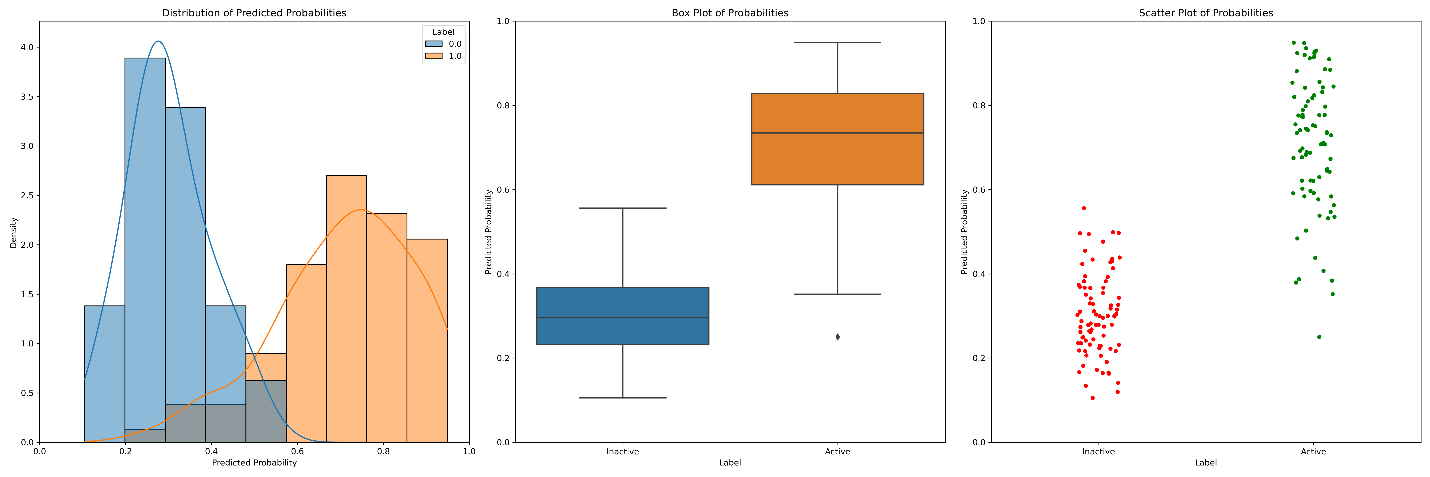


**Figure 16: Benchmarking Dataset P00644: Train and Test loss,** train loss is displayed as blue curve and test loss is depicted by orange curve. **Distribution of Probabilities,** as a histogram can be visualized having Blue (Inactive) and Orange (Active) Bars. **Box Plot of Probabilities,** determining the clear separation between active/inactive class and having a good balance between the prediction, and **Scatter Plot of Probabilities,** shows the confidence in the probabilities for both active (green) and inactive (orange) dots.


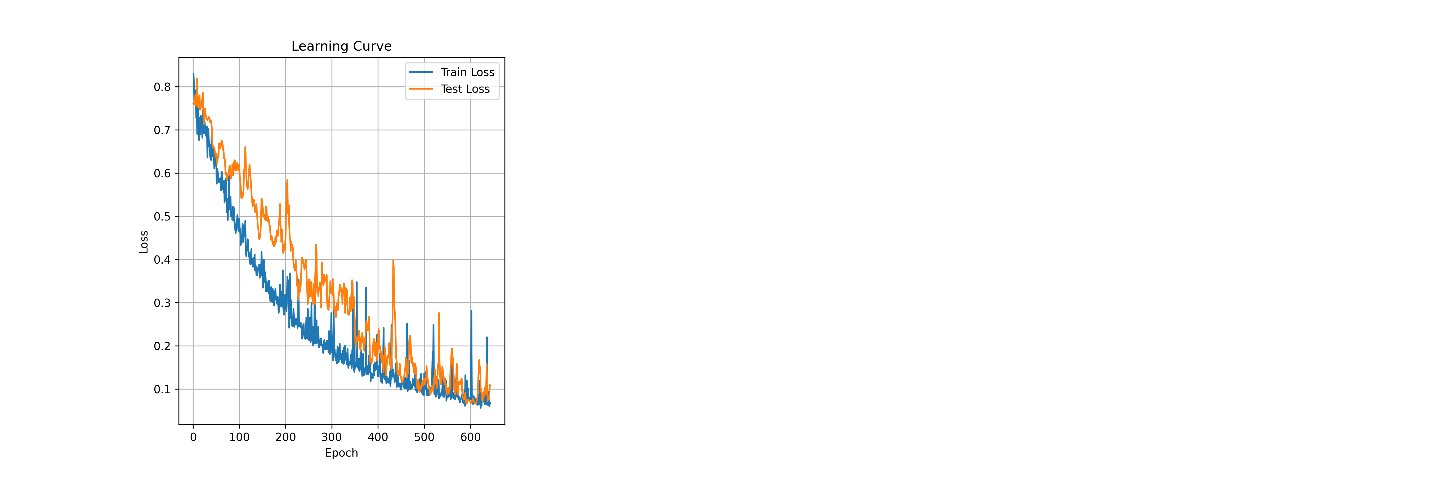

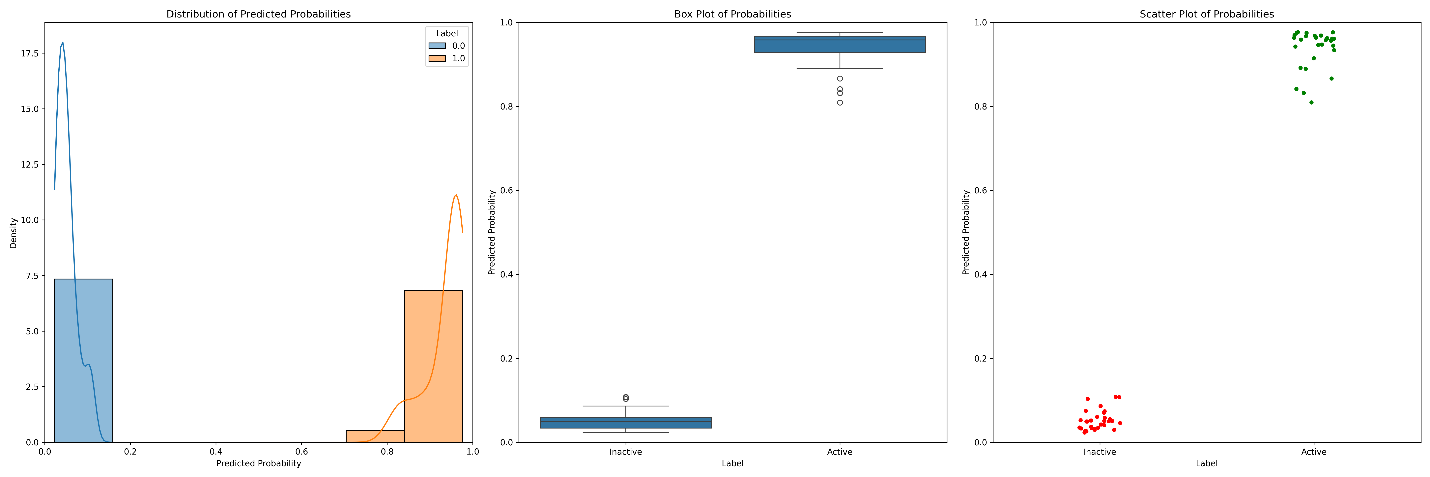


**Figure 17: Benchmarking Dataset P00648: Train and Test loss,** train loss is displayed as blue curve and test loss is depicted by orange curve. **Distribution of Probabilities,** as a histogram can be visualized having Blue (Inactive) and Orange (Active) Bars. **Box Plot of Probabilities,** determining the clear separation between active/inactive class and having a good balance between the prediction, and **Scatter Plot of Probabilities,** shows the confidence in the probabilities for both active (green) and inactive (orange) dots.


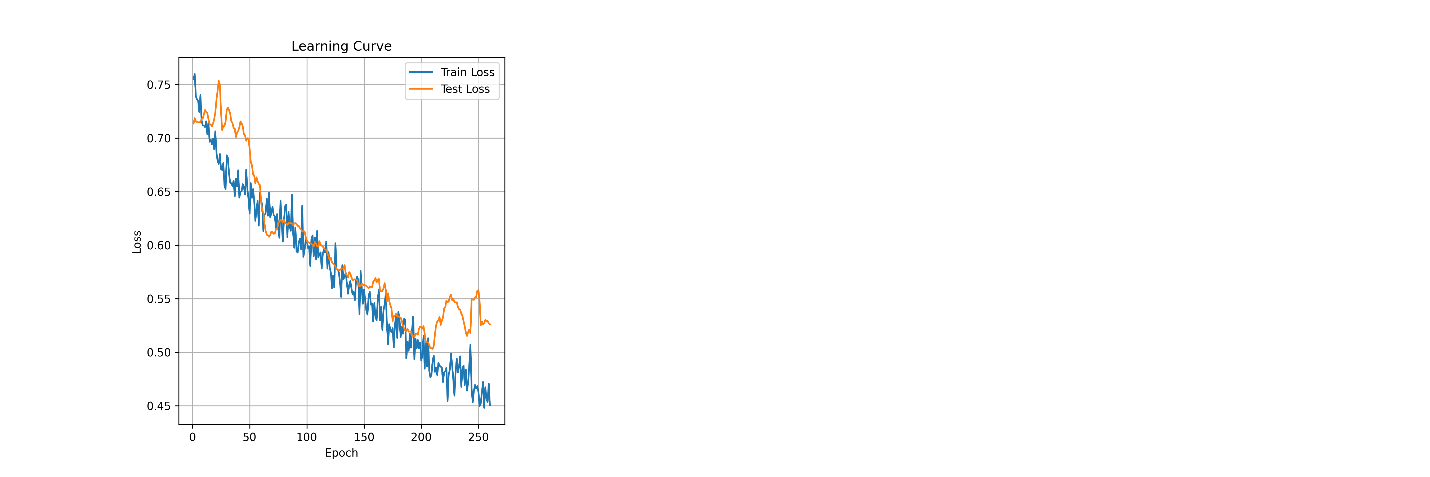

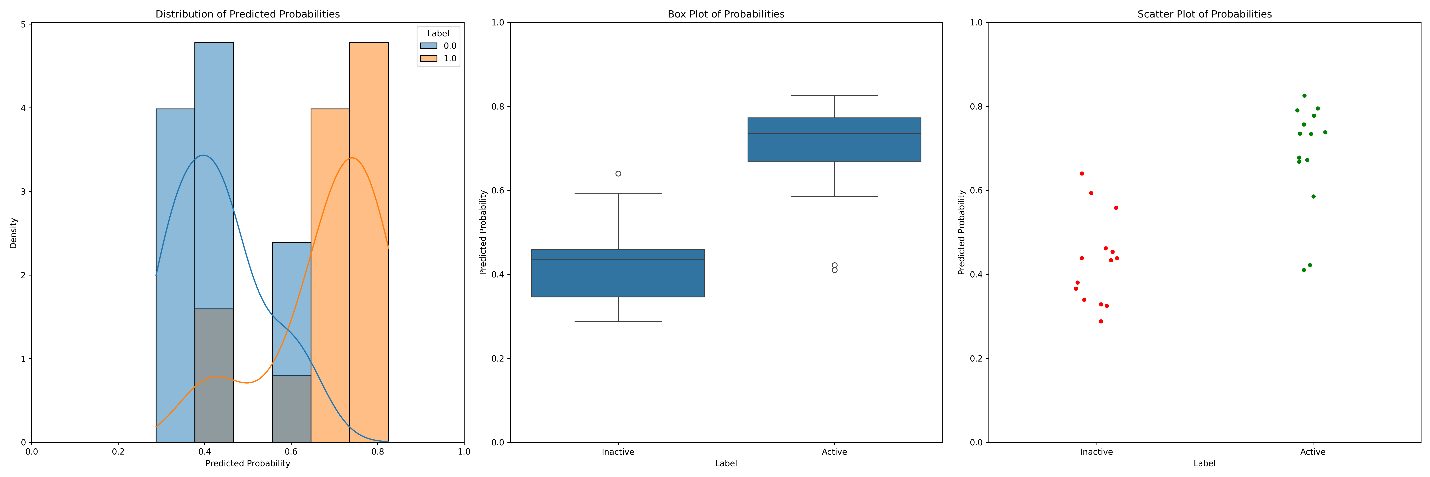


**Figure 18: Benchmarking Dataset P00720: Train and Test loss,** train loss is displayed as blue curve and test loss is depicted by orange curve. **Distribution of Probabilities,** as a histogram can be visualized having Blue (Inactive) and Orange (Active) Bars. **Box Plot of Probabilities,** determining the clear separation between active/inactive class and having a good balance between the prediction, and **Scatter Plot of Probabilities,** shows the confidence in the probabilities for both active (green) and inactive (orange) dots.


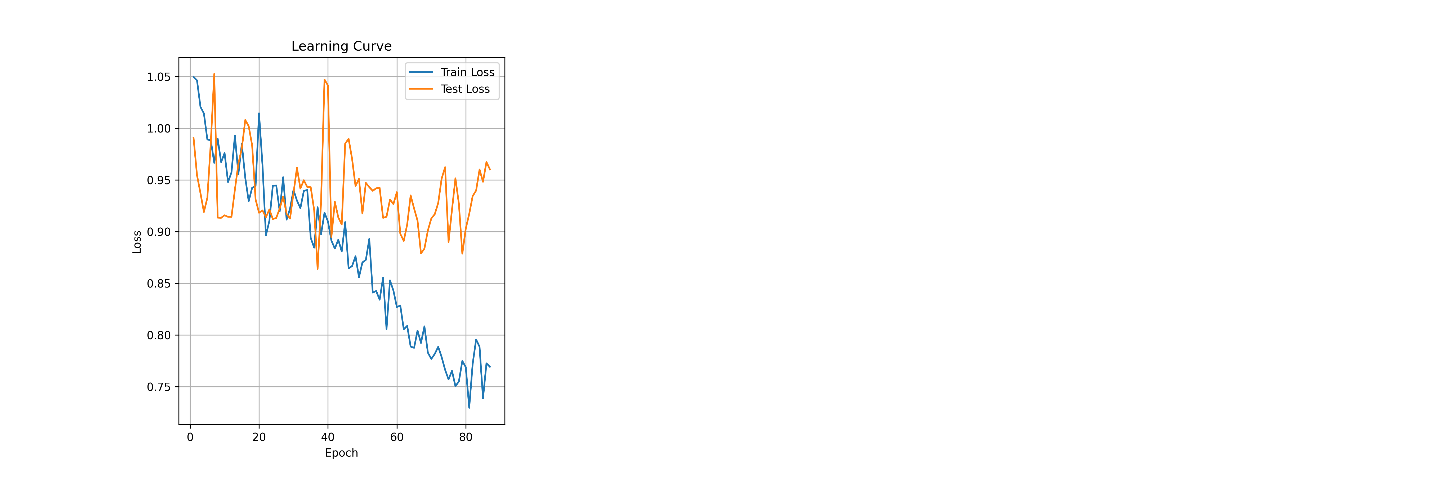

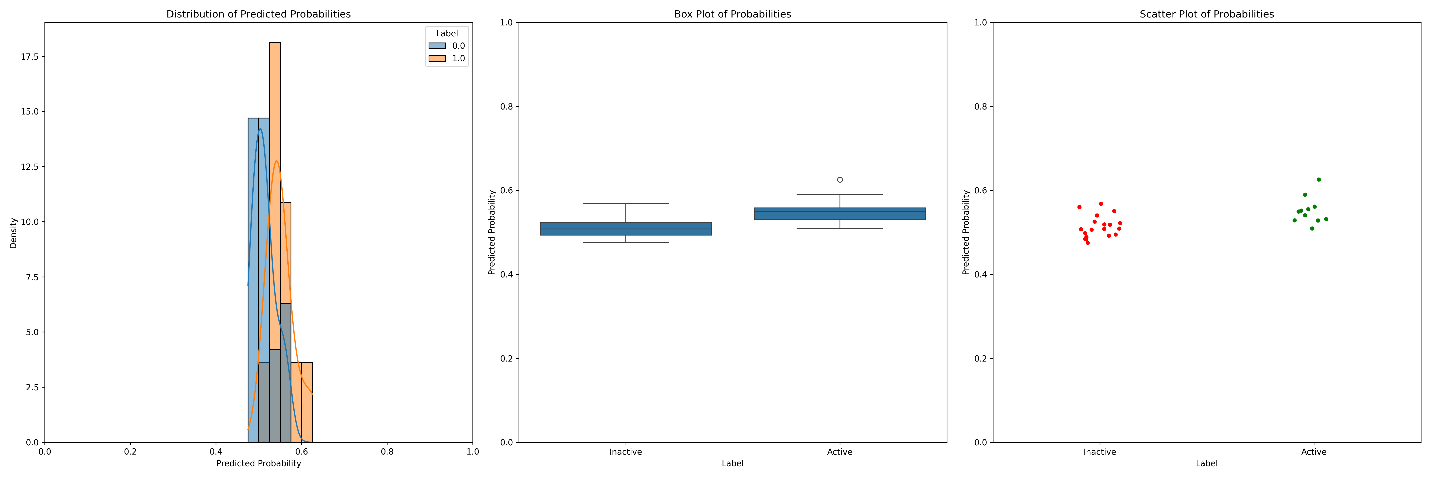


**Figure 19: Benchmarking Dataset ADGRL1: Train and Test loss,** train loss is displayed as blue curve and test loss is depicted by orange curve. **Distribution of Probabilities,** as a histogram can be visualized having Blue (Inactive) and Orange (Active) Bars. **Box Plot of Probabilities,** determining the clear separation between active/inactive class and having a good balance between the prediction, and **Scatter Plot of Probabilities,** shows the confidence in the probabilities for both active (green) and inactive (orange) dots.


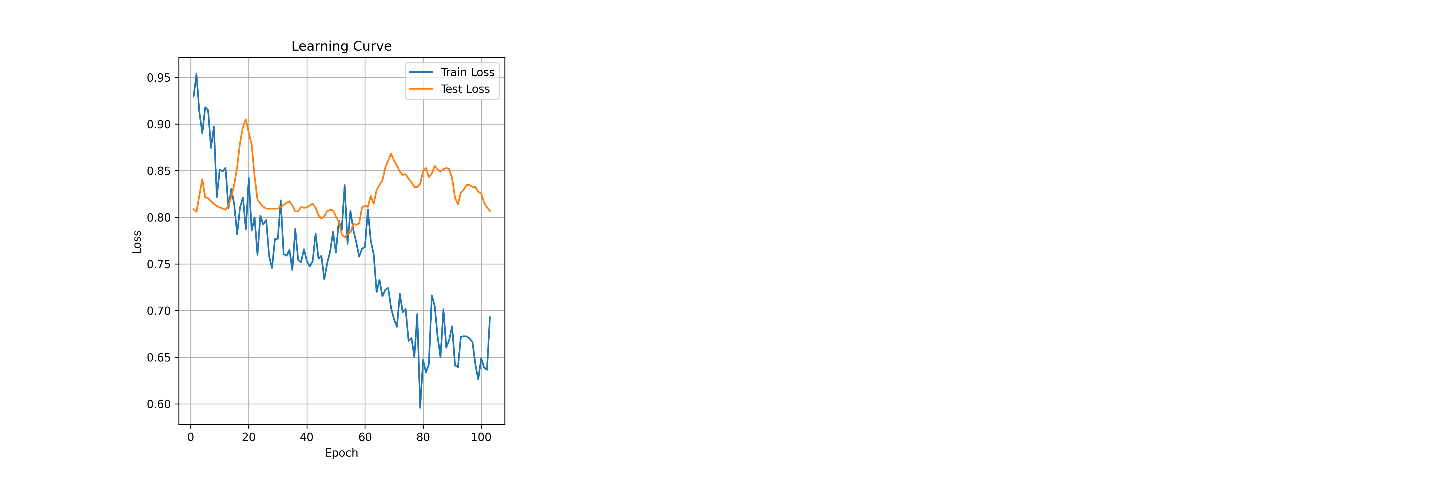

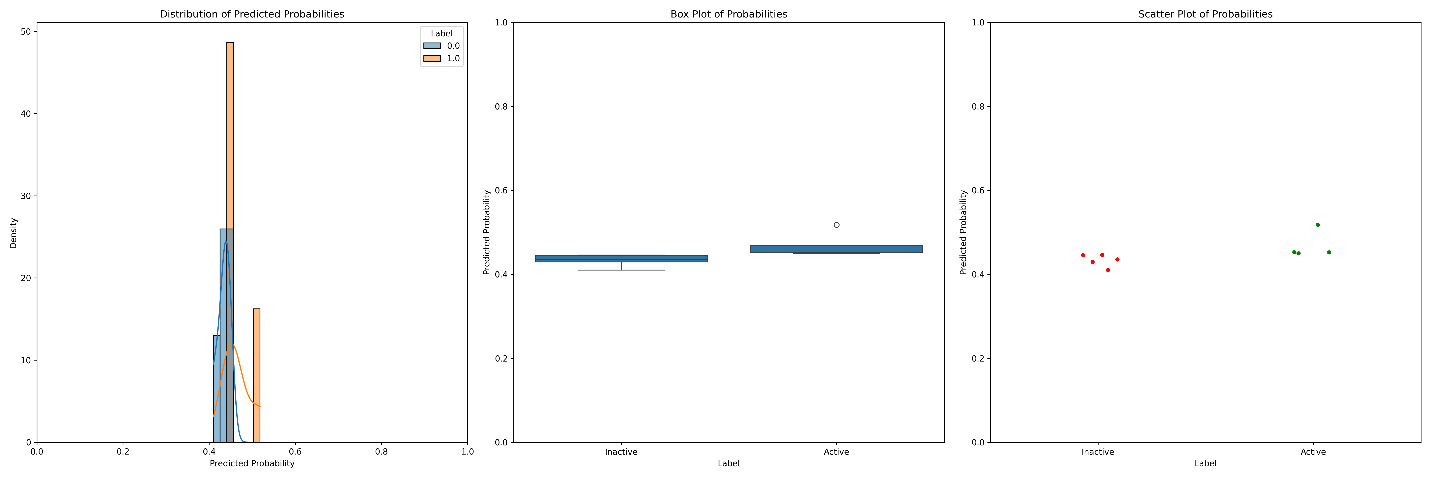


**Figure 20: Benchmarking Dataset 5H2C: Train and Test loss,** train loss is displayed as blue curve and test loss is depicted by orange curve. **Distribution of Probabilities,** as a histogram can be visualized having Blue (Inactive) and Orange (Active) Bars. **Box Plot of Probabilities,** determining the clear separation between active/inactive class and having a good balance between the prediction, and **Scatter Plot of Probabilities,** shows the confidence in the probabilities for both active (green) and inactive (orange) dots.
